# Agricultural Policies Exacerbate Honeybee Pollination Service Supply-Demand Mismatches Across Europe

**DOI:** 10.1371/journal.pone.0082996

**Published:** 2014-01-08

**Authors:** Tom D. Breeze, Bernard E. Vaissière, Riccardo Bommarco, Theodora Petanidou, Nicos Seraphides, Lajos Kozák, Jeroen Scheper, Jacobus C. Biesmeijer, David Kleijn, Steen Gyldenkærne, Marco Moretti, Andrea Holzschuh, Ingolf Steffan-Dewenter, Jane C. Stout, Meelis Pärtel, Martin Zobel, Simon G. Potts

**Affiliations:** 1 Centre for Agri-Environmental Research, University of Reading, Reading, United Kingdom; 2 Institut National de la Recherche Agronomique, Avignon, France; 3 Swedish University of Agricultural Sciences, Uppsala, Sweden; 4 Department of Geography, University of the Aegean, Mytilene, Greece; 5 Cyprus Agricultural Research Institute, Nicosia, Cyprus; 6 Department of Nature Conservation, Zoology and Game Management, University of Debrecen, Debrecen, Hungary; 7 Animal Ecology Team, Alterra, Wageningen, The Netherlands; 8 Naturalis Biodiversity Center, Darwinweg, The Netherlands; 9 Resource Ecology Group, Wageningen University, Wageningen, The Netherlands; 10 Danish Centre for Environment and Energy, Institution for Environmental Science, Aarhus University, Aarhus, Denmark; 11 Community Ecology Research Unit Swiss Federal Institute for Forest, Snow and Landscape Research, Bellinzona, Switzerland; 12 Department of Animal Ecology and Tropical Biology, University of Würzburg, Würzburg, Germany; 13 Trinity Centre for Biodiversity Research, Trinity College Dublin, Dublin, Republic of Ireland; 14 Institute of Ecology and Earth Sciences, University of Tartu, Tartu, Estonia; Temasek Life Sciences Laboratory, Singapore

## Abstract

Declines in insect pollinators across Europe have raised concerns about the supply of pollination services to agriculture. Simultaneously, EU agricultural and biofuel policies have encouraged substantial growth in the cultivated area of insect pollinated crops across the continent. Using data from 41 European countries, this study demonstrates that the recommended number of honeybees required to provide crop pollination across Europe has risen 4.9 times as fast as honeybee stocks between 2005 and 2010. Consequently, honeybee stocks were insufficient to supply >90% of demands in 22 countries studied. These findings raise concerns about the capacity of many countries to cope with major losses of wild pollinators and highlight numerous critical gaps in current understanding of pollination service supplies and demands, pointing to a pressing need for further research into this issue.

## Introduction

Insect pollination is an important ecosystem service to agriculture, improving production in ∼75% of global crops [1], including many important sources of nutrients in the human diet [2], and contributing an estimated €153bn to global agricultural crop value [3]. Globally the area of insect pollinated crops has increased >300% since 1961 [4] and value added by pollination services is an increasingly important component of agricultural GDP in many nations including the USA and Russia [5], greatly increasing the need for secure, stable supplies of pollination services. Among the numerous species that provide pollination services, the eusocial, generalist Western honeybee (*Apis mellifera*) is reported to visit the greatest variety of crop species [1]. Although honeybees are readily managed for pollination service provision in much of the world, recent studies suggest that diverse wild pollinator communities often provide equal, superior or complementary service levels to managed honeybees [6], [7]. Recent studies have demonstrated widespread declines in wild pollinator diversity across much of Europe due to a combination of agricultural intensification, habitat degradation, the spread of diseases and parasites and climate change [8]. Furthermore, due to the absence of dedicated monitoring schemes, little information exists on the stocks and flows of wild pollinators [9]. By contrast, although honeybee stocks have suffered severe declines in many parts of Europe, due largely to the spread of parasites and rising beekeeping costs [10] they remain more resilient to habitat and resource declines than wild pollinators [11]. Managed honeybee populations are also monitored on a regular basis, providing insight into trends and stocks. As such, even where they are not principal pollination service providers, ample managed honeybee stocks can provide insurance against wild pollinator losses or fluctuations.

While a number of studies have examined the drivers and economic consequences of pollination service declines [3], [5], little attention has been given to available service supplies relative to demands. While it is difficult to assess supplies of wild pollinators, managed honeybee colony numbers are often recorded, thereby allowing for a comparison of honeybee supply relative to service demands. Globally, honeybee stocks have risen at a slower rate than the growth in planted area of insect pollinated crops [4]. Within Europe, recent reforms to the common agricultural policy have removed production linked subsidies and relaxed market price controls resulting in significantly increased farmgate prices for many subsidised crops, notably oilseed rape where prices have risen by an average of 65% between 2005 and 2010 [12], [13]. Demand for oilseed crops has been further increased following the introduction of the renewable fuel directive in 2003 which required liquid biofuels to form 5.75% of transport fuel consumption in member states by 2010 [14]. Recent research has demonstrated strong links between this policy and significantly increased planted areas of biodiesel feedstocks, such as soybean, oil palm and oilseed rape, both across Europe [15] and globally [16], [17]. How these changes relate to demand for pollination services remains unclear due to varying crop requirements for pollination services [18]. Using official data from national authorities, this study assesses the impacts of changes in crop agriculture and honeybee stocks between 2005 and 2010 on the maximum capacity for honeybees to act as the sole supplier of pollination services for 41 European countries. Those countries with a low stock of honeybees are likely to be more reliant upon wild pollination services to meet their demands than other countries, although it is not within the study's capacity to estimate the actual contribution of either group to national service supply, only the capacity of honeybee stocks to do so under ideal conditions.

## Methods

### Crop and Honeybee Data

Sufficient data was available for 41 countries viable for inclusion in this study including all current EU members. Supplemental S1 contains details of all sources used and any specific transformations and assumptions used. Countries were allocated into regions [19] with Armenia, Georgia and Cyprus included in Southern Europe. National agricultural statistic data were used as primary data sources as, unlike multinational databases, these are often subject to revision and can contain a broader range of crops (e.g. caraway, a major crop in Finland). FAO data also contains several significant inaccuracies, notably suggesting that Belarus and Latvia have <100 beehives each. Cucumbers and peppers were only included for Southern European countries or where they were explicitly stated as being grown in the open as they are otherwise grown in glasshouses where honeybees are not commonly employed [18]. Tomatoes, eggplants, linseed and groundnut were also excluded either because they require buzz pollination to produce seeds or because pollination has little to no benefit to yields [1]. For EU members which do not record honey bee colony numbers annually, 2010 numbers were taken from annex I of Commission Regulation (EU) No 726/2010 as the most recent data available for these countries, although it should be noted that member states were under no obligation to collect this data in a standardized manner or at the same time. For Norway, where no 2010 honey bee data could be acquired, it was assumed that stocks have remained constant since 2005.

### Recommended Stocking Rate (RSR) Values

Demand for managed honeybee pollination services can vary between crops, requiring different numbers of honeybees to provide adequate pollination services. As such, recommended stocking rates (RSR) from published literature (Supporting Information S2) were used to estimate each crops demand for pollination services. To capture uncertainty, three RSR values were used for each crop; lower and upper, representing the minimum and maximum values found in the literature respectively, and average representing the mean value of all values reported in the cited literature. Where crop specific estimates were not available, a closely related crop was used as a proxy. If no closely related crop was available, then the mean values of similar crops or those with similar floral morphology were used.

### Supply Density

Honeybee stocks strongly correlate with country size, resulting in larger countries having greater stocks. Consequently, available supply of honeybee colonies was compared between countries using potential Supply Density (SD) of honey bee colonies available per hectare of insect pollinated crop.
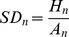
(1)Where *SD_n_* is the supply density of honey bee colonies in country n, *H_n_* is the total number of honey bee colonies available and *A_n_* is the total area of insect-pollinated crops, excluding those that cannot be pollinated by honeybees. Although varieties of some crops can be entirely self-fertile, thereby requiring no additional pollination from insects to produce maximum yields, the extent to which these varieties are used is largely unknown. Therefore the whole area of each crop was assumed to require insect pollination.

### Total Demand and Density of Demand

The total number of honeybee colonies required to provide adequate pollination services in each country is estimated as:
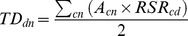
(2)Where *A_cn_* is the area of crop c in country n and *RSR_cd_* is the recommended stocking rate of honeybee colonies required per hectare of crop *c* to provide adequate pollination services under assumption *d* and is divided by two to represent the capacity for honeybee hives to be moved once between crops within a year. More than two moves are possible, but considered unrealistic in many countries and can prove complex to account for different crop phenology in large, climatically varied countries such as France. National demand for pollination services is the product of the area of insect-pollinated crops and the recommended stocking rate of honey bee colonies per hectare of these crops. As the area of insect-pollinated crops and, by extension, demand for pollination services is strongly linked with total country size (i.e. large countries will have higher demands than smaller ones), comparison of demand between countries is expressed through density of demand, the weighted average of honey bee colonies required per hectare of insect-pollinated crops
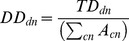
(3)


### Pollination Service Capacity

The maximum Pollination Service Capacity (*PSC*) of honeybee stocks to provide adequate pollination services to crops in each country, regardless of wild insect availability, was estimated by dividing the supply density by density of demand under each of the three RSR assumptions.
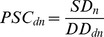
(4)Where *PSC_dn_* is the pollination service capacity, under *RSR* density d, of honeybee stocks in country n. This is equivalent to the total number of honeybee colonies divided by half the total number of colonies demanded. This method inherently assumes that all hives are managed effectively for pollination services with no overstocking and are moved once per year between crops which require pollination. It must be noted that this is unlikely to be the case as in many European countries limited markets for pollination services presently exist and many beekeepers are amateurs or exclusively concerned with honey production [10]. As such it represents a “best case” scenario of the maximum possible contribution of honeybees to crop pollination.

### Statistical Analysis

Data were assessed for normality using Shapiro-Wilk tests. Relationships between continuous variables were assessed using Pearson's product moment correlation coefficient (r) or Spearman's Rank Correlation coefficient (*ρ*) for non-normally distributed density of demand and change in density of demand variables. The significance of geographic variations in annual Supply Density, density of demand and Pollination Service Capacity and changes in these variables were assessed using categorical regression models with factor variables for EU membership and Northern or Southern Europe. Density of demand values were Log transformed to normalise their distributions. All analyses were conducted in R [20]. Supporting Information S3 contains the full results of these analyses. Greece was excluded from all analyses involving the relative change in national biofuel area due to its extremely high relative growth acting as an outlier.

## Results

### Total Stocks, Area and Demand

**Figure 1:**
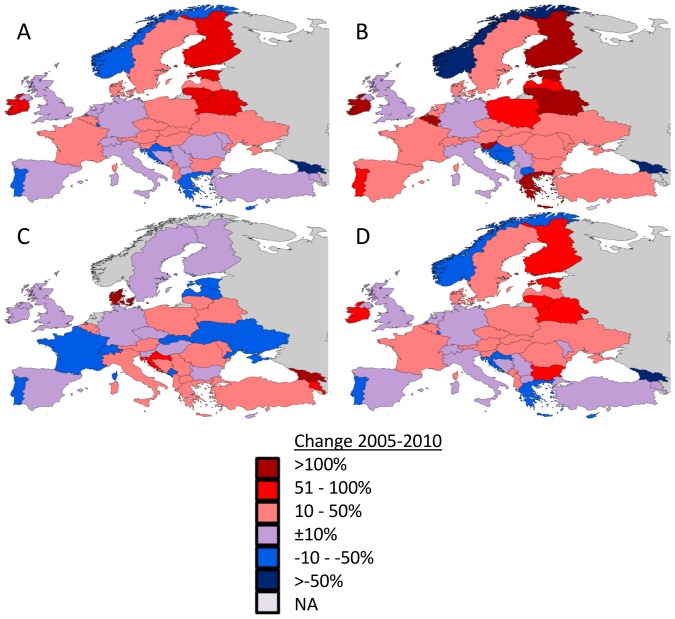
National percentage change in total honeybee stocks (A), the total area of insect pollinated crops (B), the total national area of three main biofuel crops (oilseed rape, sunflower and soybean) (C) and the total number of honeybee colonies required to provide adequate pollination services under average RSR assumptions (D) between 2005 and 2010.

Total honeybee stocks across the 41 countries rose by 7% between 2005 and 2010 from 22.5 M colonies to 24.1 M colonies, with stronger increases in southern European countries where beekeeping is more common (Figure 1a). Although national stocks more than doubled in Georgia, Denmark and Malta, 15 countries experienced declines of between 4% (Slovenia) and 47% (Switzerland). In both years ∼45% of European honeybee stocks were located across Turkey, Ukraine and Spain. Overall area of crops pollinated by honeybees increased by 17% from 23.1 M ha to 27.1 M ha; 2.2 times the rate of honeybee stock increases in the same period. Pollinated crop area increased in most (32) countries (Figure 1b) but was particularly high in northern European countries such as Finland (91%) and Lithuania (70%). Some, mostly southern European countries saw significant area contractions, notably Georgia (−62%) and Cyprus (−39%).

Much of the observed increase in pollinated crop area was driven by growth in insect pollinated biofuel feed crops (oilseed rape, sunflower and soybean), planted area of which collectively rose by 4.2 M (32%) ha across 38 of the countries studied. The absolute increase of these crops was greatest in Ukraine (2.1 M ha), although Greece saw the highest proportionate increase (717%) (Figure 1c). Changes in biofuel crop area were significantly greater in EU member countries than countries outside the union, although this was only significant in older EU states (β = 0.487, p = 0.02), and there was a strong correlation between changes in biofuel area and overall changes in insect pollinated crop area (r = 0.79, p<0.001) (Supporting Information S3). Only five countries saw decreases in planted areas of biofuel crops, most significantly Georgia where total area of these crops fell by 29,177 ha (75%). It should be noted that the actual use of these crops for biofuel feedstock is unknown as few countries report area specifically grown for this purpose and biofuel producers can source additional feedstock from the market. A range of recommended stocking rates (RSR) of honeybee colonies required per hectare of individual crops was used to estimate national demands for pollination services. Using the literature average RSR values for each crop, the total number for honeybee colonies required to meet pollination service demands across all 41 countries rose by ∼9 M, 4.9 times the actual rate of honeybee stock growth. Changes in total demand strongly reflected changes in the total area of insect pollinated crops, with the greatest increase in Finland (71%, 0.7 M colonies) and decrease in Georgia (−56%, −0.2 M colonies) respectively (Figure 1d).

### Supply and Demand Density

**Figure 2:**
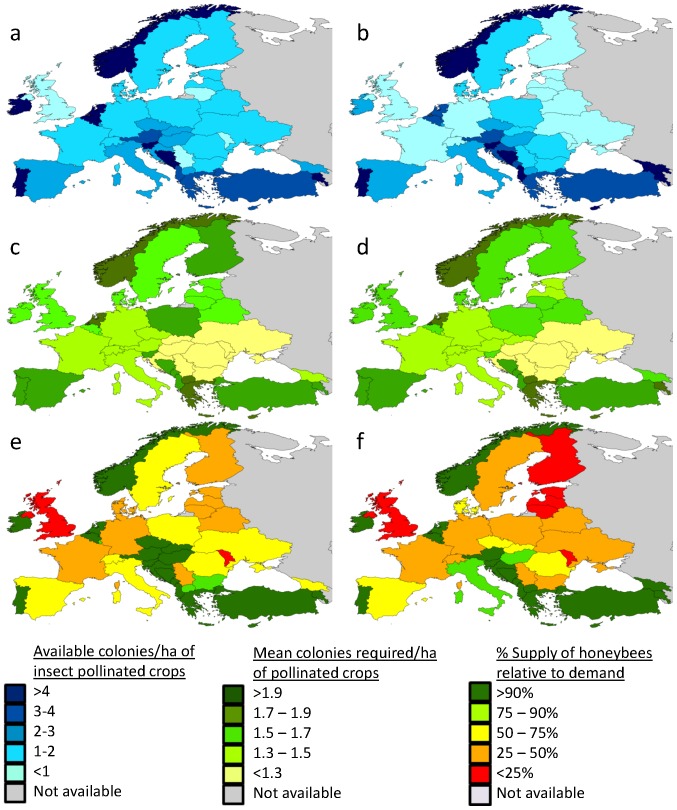
A comparison of the supply density of honey bees (a, b), density of demand (c,d) and the resultant pollination service capacity (e,f) in 2005 (left panels) and 2010 (right panels). Figures based on average recommended stocking rate (RSR, see Supporting Information S4 for figures and comparative discussion based on other RSR).

In both years (Figure 2a,b), the relative availability of honeybee colonies per hectare of insect pollinated crops (Supply Density – SD) was highest in Slovenia (12.5 and 8.9 colonies/ha respectively) due to topographical conditions limiting available cropping area. The lowest SD was found in Moldova (0.2/ha) where oilseed crops occupy much of the farmed landscape but honeybee stocks remain relatively low. Between 2005 and 2010, average national SD rose by 12%, however, this is upwardly biased by >100% SD increases in Georgia, Croatia and Malta where stocks have risen significantly while pollinated crop area has fallen or remained stable. Without these three countries, national SD has fallen by an average of 5% due to either falling honeybee stocks, rising total crop area or both of these factors with >25% declines in 15 countries (Supporting Information S5).

In both years density of demand (DD), the weighted average number of colonies required per hectare of insect pollinated crop was negatively influenced by the ratio of oilseed crop area to orchard fruits (*ρ* = −0.63 and −0.64, p<0.001), the latter typically demanding more colonies per hectare (Supporting Information S2). Consequently, the Netherlands (2.1 colonies/ha), which has very little oilseed crop area, had the greatest DD under average RSR in both years (Figure 2c,d). Unlike SD, DD did not vary by more than 10% between years in most countries, falling on average by 2% (0.03 colonies/ha).

### Pollination Service Capacity

Analysis of the capacity of national honeybee stocks (PSC) to supply demands indicates that, under average RSR assumptions, there were honeybee deficits (insufficient stocks to supply ≥90% of national demands) in 23 countries in 2005 and 22 in 2010, two of which (UK and Moldova) had PSC below 25% (Figure 2e,f). Between 2005 and 2010, four countries (Luxembourg, Malta, Macedonia and Georgia) moved out of deficits due to greater SD, while falling SD in three more (Czech Republic, Slovakia and Hungary) pushed PSC below 90%. Of the 19 countries (47% of those studied) experiencing deficits in both years, PSC on average fell by 18% and four more countries fell below 25% PSC. Five countries also had <90% PSC under even lower RSR assumptions, rising to 11 countries by 2010 (Supporting Information S3). Taken as a single region, under average RSR, European PSC falls from 66% to 64% between the two years.

Correlation analysis in R indicates that SD was more strongly correlated with DD in 2010 (*ρ* = 0.59, p<0.001) than in 2005 (*ρ* = 0.48, p<0.001), mostly due to falling SD in many countries with already low DD (e.g. Ukraine). Although this strengthening relation suggests that honeybee stocks may be influenced by demand for pollination services, there was no correlation between percentage changes in SD and changes in DD (*ρ* = 0.06 p = 0.723), or PSC in 2005 (r = −0.07, p = 0.623), indicating that stocks did not respond to changes in relative demand or existing service capacity as would be expected if honeybees were actively managed for pollination services. Annual PSC was more strongly correlated with SD (r = 0.99, p<0.001 both years) than DD (*ρ* = 0.36, p = 0.022 in 2005 and *ρ* = 0.47, p = 0.002 in 2010). This relationship derives from the influence of southern European countries where climatic conditions facilitate abundant beekeeping and cultivation of fruit crops which have greater honeybee colony demands per hectare. Conversely, PSC was not correlated with honeybee stocks (r = −0.11, p = 0.511 in 2005 and r = −0.06 p = 0.687 in 2010) or area of insect pollinated crops (r = −0.23, p = 0.141 in 2005 and r = −0.28, p = 0.071 in 2010). There was a strong negative correlation between relative changes in biofuel area and both PSC (r = −0.53, p<0.001) and SD (r = −0.52, p<0.001), confirming that, despite the low numbers of colonies required to provide adequate pollination per hectare, large scale oilseed expansion has substantially reduced national PSC by lowering SD, even in countries where honeybee stocks have increased.

## Discussion

### Supply and Demand for Pollination Services

This study utilises data from 41 European countries to examine the supply and demand for honeybee pollination services and evaluates the changing capacity of honeybees to provide these services. The results of this study highlight the growing importance of pollination services as an agricultural input across Europe, with demand for honeybee pollination services rising 4.9 times as fast as available stocks. In many countries, this has caused the availability of honeybees relative to insect pollinated crop area to fall substantially. These trends are driven by substantial increases in oilseed crop cultivation and with this, demand for pollination services. Much of the expanded area of biofuel crops has come at the expense of barley and other cereal crops in response to market price increases that have arisen from a combination of relaxed EU price controls and rising demand for biodiesel to meet renewable fuel targets [12]. Further increases in cultivation of biofuel crops across Europe in response to growing biofuel crop demands as member states increase consumption towards a proposed 10% target for 2020 [21], are expected to further increase the disparity between supply and demand for pollination services. Changes in national or international agricultural policy, such as encouraging greater European production of insect pollinated livestock feeds could further increase demands. Alternatively, a greater utilization of non-oilseed biofuel feeds (e.g. miscanthus) and land taken out of production, should certain greening requirements be adopted by a revised CAP, [22], [23] may reduce overall demand. Consequently, this study points to an immediate need for substantial research into the pollination service demands in the main cultivars of Europe's major crops. Ideally, such efforts should be accompanied by wide scale monitoring of pollination service delivery to determine what, if any, yield gap exists as a result of inadequate pollination.

Analysis of the pollination service capacity of national honeybee stocks indicates that 22 countries have insufficient colonies to supply >90% of the pollination service needs and that service capacity has fallen across most countries. While these findings do not in themselves indicate the relative importance of honeybees and wild pollinators, they do demonstrate an increasing reliance upon wild pollination services across much of Europe. Unlike honeybees, the status and trends of wild pollinators remains largely unknown, although recent studies have indicated significant losses of wild pollinator diversity [8], much less is understood about the abundance of these insects due to limited monitoring efforts [9]. Furthermore, even in countries where honeybees are readily available, wild pollinator communities may be substantial contributors to actual service delivery [6], [7] and synergistic interactions between wild pollinators and honeybees have been demonstrated to enhance pollination in several crops, including almond [24], strawberry [25] and hybrid sunflower [26], limiting yield in the absence of either group. Consequently, countries with low honeybee PSC are potentially more vulnerable to negative shifts in wild pollinator communities. This result is of particular concern as many of these countries also have limited availability of good quality wild pollinator habitat [27]. Nonetheless even in a state of decline wild pollinators may be able to support crop pollination services due to a range of ecological shifts. First, most evidence of wild pollinator declines derives from falling species diversity, however there is less evidence of declining pollinator abundance [28], subsequently services may have been maintained through the growing dominance of more resilient species. A recent study by Carvalheiro et al. [29] suggests this may be the case in the UK, the Netherlands and Belgium, with localised pollinator communities becoming more homogenised as wider diversity declines. Second, high species diversity may allow for functional redundancy [30] as long as key traits are not lost (e.g. [31]). Third, mass flowering crops may act as a resource sink for wild pollinators, regardless of honeybee density, resulting in ample pollination even with declining populations [7], [32], [33]. These uncertainties, as well as the uncertainties inherent in which groups are actually responsible for pollination service provision highlight a growing need for further research into the provision of pollination services across Europe. This is particularly important in several low GDP countries such as Moldova, Ukraine and Romania where insect pollinated crops occupy a high proportion of crop area and agricultural production forms an important component of GDP [34], [35].

### Knowledge Gaps and Uncertainties

Understanding of the pollination service demands of crops is presently very limited, as evidenced by the wide range of recommended stocking rates (RSR) of honeybees within some crops in this study. This in turn limits the capacity to project policy impacts on service demands at different spatial scales. These RSR estimates are inconsistently estimated and unlikely to be broadly applicable; for instance the lowest RSR for oilseed rape (1 colony/ha) is taken from a field study in Australia [36] where feral honeybees among other wild pollinators are widespread and thus greater “ambient” services are available. By contrast a more comprehensive study from Canada, where climates are similar to much of Europe, demonstrates that 3 colonies/ha provide significantly greater yield than 1.5 colonies/ha [37]. Furthermore, for some crops, existing RSR values are very limited, such as soybean for which only a single estimate is available [18]. These shortcomings can be overcome with standardized studies undertaken as part of cultivar trials [38] including a wide variety of potential pollinators, particularly for crops where honeybees are sub-optimal pollinators, such as field beans [39]. Nonetheless, the use of RSR values in this study represent a more realistic measure of demand than the coverage of insect pollinated crops alone, due to the widely observed differences in densities required between crops.

The findings of this study are based upon several assumptions that may exaggerate or reduce the estimated pollination service capacity of national honeybee stocks. Foremost, PSC values are very sensitive to the assumption that only insect pollinated varieties are utilised, particularly in the case of oilseed rape given its wide geographic coverage [40]. However recent research has demonstrated that yields of self-fertile lines of oilseed rape still benefit significantly from insect pollination in field conditions [41] and, lacking detailed agronomic assessments of the pollinator dependence of current major cultivars, it is unlikely that the results presented significantly over-estimate service demand. By contrast, the availability of honeybee stocks is likely to be overstated in several countries as most beekeepers are either hobbyists or exclusively concerned with honey production (e.g. [10], [42]). As such, it is unlikely that these beekeepers will either deliberately place their colonies near crops or move them between crops (e.g. [43]). Similarly, the location of beekeepers may not correspond to the location of crops, resulting in regional service deficits even if national stocks are theoretically sufficient. Where honeybees are used for pollination services, lacking consistent information on the number of colonies required or specific management requirements [e.g. 44], [45] it is possible that they may be over or under stocked [43].

Supplies of pollination services are similarly poorly understood. Although a number of studies have identified the relative contribution of different insects to pollination services in several landscapes [6], [7], [32], [46] data on the overlap of principle pollinators and crops remains limited (but see [19]). This limitation could be overcome via systematic monitoring of pollinator diversity and abundance, [10] combined with analysis of the relationship between the floral traits of crops and the functional traits of effective pollinators, allowing for more accurate estimation of service availability. Monitoring service delivery is also possible using standardised hand pollination and bagging experiments [38], however this would not identify the causes of declining supply, only the extent of supply shortfalls and yield limitations.

### Conservation Actions

Although the findings of this study do not infer either yield losses or the relative importance of wild pollinator or honeybees, they do nonetheless highlight those countries which are inherently more likely to rely upon wild pollination services. As such, the findings raise questions regarding the potential for proposed conservation actions to mitigate some of this risk and highlight the need for accounting for both groups. While over-reliance upon managed honeybees can result in substantial price spikes should populations crash [47], [48], reliance upon wild pollinators may be unsuitable for large scale agriculture due to their less predictable numbers and vulnerability to stochastic shocks [46]. Unlike other agricultural inputs, pollination services are affected by a number of environmental, social and economic factors rather than a controlling market and subsequently require a multi-faceted approach rather than a single policy solution.

Although honey markets are well established, there are limited markets for honeybee pollination services, despite the potentially significant value of this service to producers [3]. Furthermore, honeybee populations remain under pressure from climate change, new pests such as the Asiatic hornet (*Vespa velutina*) [49], and pesticide exposure [50], [51] (but see [52]). Efforts to reduce beekeeping costs could be achieved by improving access to effective Varoosis medication, which is presently limited in much of Europe [53], supporting national bee health plans, such as those present in EU [54] and encouraging beekeepers to expand and diversify their activities through rural development funding (e.g. [55]). Another option is the broader use of managed pollinators, such as the buff-tailed bumblebee (*Bombus terrestris*) and the red mason bee (*Osmia bicornis*) can provide superior services to honeybees for specific crops [6] and new species could potentially be domesticated to provide optimised, crop-specific service delivery [56]. While markets for these alternative pollinators are growing annually, the unregulated use of any managed pollinators can result in disease spillover [57], [58], outbreeding [59] and resource competition with wild bees [60], [61]. These issues can potentially be mitigated through the use of native rather than imported subspecies as breeding stock and careful disease screening.

Wild pollination services are closely associated with pollinator diversity with beneficial synergies [23] and redundancies emerging from diverse assemblages [30]. Wild pollinators continue to face pressures from declining resource availability [8] and increasing agrochemical use in several countries including the UK, Germany and Hungary [62], [63]. Large scale mass flowering crops can increase wild pollinator populations [64], [65] (but see [66]), although additional resources may need to be provided to ensure sufficient forage after the initial resource pulse [67] (but see [68]), and may reduce pollination to native plants [33] or increase competition within communities later in the season [69]. Consequently, wild pollinators benefit from crop diversification, agri-environment measures that increase resource diversity and reduced inputs in key flower-rich habitats [70]. The uptake of these measures may in turn be limited by costs and the complexity of implementation [71] and cultural resistance from farmers [72], [73]. In some cases there may be a significant lag in the occurrence of benefits; for example plant diversity can take >20 years to recover from the effects of 10 years intensive inputs [74]. However, these measures can also provide additional ecosystem service benefits such as nutrient cycling and biological pest control [75], [76]. The evolution of these schemes, particularly in newer EU members, could therefore increase ecosystem service security by providing new measures that better fit with changing agricultural practices. Finally, it will be essential to demonstrate the full costs and benefits of such measures to productivity.

## Supporting Information

Supporting Information S1 **Data Sources.**
(DOCX)

Supporting Information S2 **Recommended Stocking Rates (RSR) of honeybee colonies per hectare of crops.**
(DOCX)

Supporting Information S3 **Full Analytical results.**
(DOCX)

Supporting Information S4 **Analysis under lower and upper Recommended Stocking Rates.**
(DOCX)

Supporting Information S5 **Summary of National Scale Results.**
(DOCX)
