# Correction: Agricultural Policies Exacerbate Honeybee Pollination Service Supply-Demand Mismatches Across Europe

**DOI:** 10.1371/journal.pone.0091459

**Published:** 2014-02-28

**Authors:** The PLOS ONE Staff

The following information was missing from the funding section: BBSRC, DEFRA, NERC, the Scottish Government and the Wellcome Trust, under the Insect Pollinators Initiative crops project. The funders had no role in study design, data collection and analysis, decision to publish, or preparation of the manuscript.

Figures 1a, 1b and 1c are in the wrong order. Here are the corrected Figures 1a, 1b and 1c.

**Figure 1:**
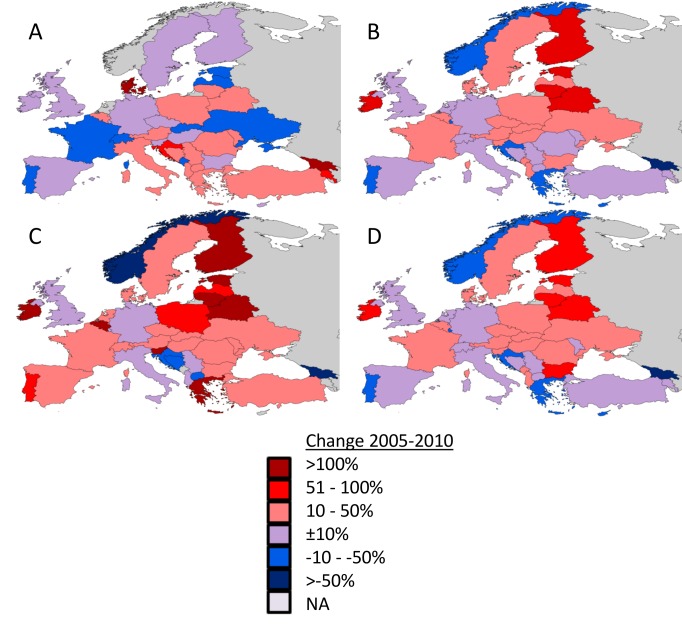
National percentage change in total honeybee stocks (A), the total area of insect pollinated crops (B), the total national area of three main biofuel crops (oilseed rape, sunflower and soybean) (C) and the total number of honeybee colonies required to provide adequate pollination services under average RSR assumptions (D) between 2005 and 2010.
