# Supplementary material for: Agricultural Policies Exacerbate Honeybee Pollination Service Supply-Demand Mismatches Across Europe
Source: PLoS One. 2014 Jan 8;9(1):e82996. doi: 10.1371/journal.pone.0082996 (PMC3885438; doi:10.1371/journal.pone.0082996)
Supplement: Supporting Information S1 — Data Sources. (DOCX) [file pone.0082996.s001.docx]

**Supplemental S1 – Data Sources**

***Albania*** *Honeybees*Republic of Albania Institute of Statistics (2012) *Number of Livestock* <http://www.instat.gov.al/en/figures/statistical-databases/select.aspx?rxid=8b9420bc-f23f-428e-b005-94037cd86317&px_tableid=BU0007> accessed 11/11/13

*Crops*Republic of Albania Institute of Statistics (2012) *Structure of Field by Crop Plantings* <http://www.instat.gov.al/en/figures/statistical-databases/select.aspx?rxid=341a4173-fc93-4f6d-b38f-6526266f121b&px_tableid=BU0020> accessed 11/11/13

FAOStat (2013) *Crops* <http://faostat.fao.org/site/567/DesktopDefault.aspx?PageID=567#ancor> accessed 11/11/13, last updated 08/08/13

***Armenia*** *Honeybees*National Statistical Service, Republic of Armenia (2012) *Honeybee numbers* Data available on request from <http://www.armstat.am/en/>

*Crops*FAOStat (2013) *Crops* <http://faostat.fao.org/site/567/DesktopDefault.aspx?PageID=567#ancor> accessed 11/11/13, last updated 08/08/13

***Austria*** *Honeybees*in Potts S.G., Roberts S.P.M., Dean R., Marris G., Brown M.A., Jones R., Neumann P. and Settele J. (2010b) Declines of Managed Honeybees and Beekeepers in Europe; *Journal of Apicultural Research 49*, 15-22

European Commission (2010) *Commission Regulation (EU) No 726/2010* <http://eur-lex.europa.eu/LexUriServ/LexUriServ.do?uri=OJ:L:2010:213:0029:0030:EN:PDF> accessed 11/11/13

*Crop Area and Yield*
Statistik Austria (2013) *Agrarstrukturerhebung 2010 – Viehbestand* <http://sdb.statistik.at/statistik.at/ext/superweb/loadDatabase.do?db=deas1002> accessed 11/11/13 Last Updated 02/08/13

***Belarus*** *Crops and Honeybees*National Statistical Committee of the Republic of Belarus (2010) *Agriculture of the Republic of Belarus 2010* <http://belstat.gov.by/homep/en/publications/agro/2010/main.php> accessed 11/11/13

*Crops*FAOStat (2013) *Crops* <http://faostat.fao.org/site/567/DesktopDefault.aspx?PageID=567#ancor> accessed 11/11/13, last updated 08/08/13

***Belgium*** *Crops*Directorate of General Statistics and Economical Information (2011) *Recensement agricole de mai 2010* (in French) <http://economie.fgov.be/fr/modules/publications/statistiques/economie/enquete_agricole_de_2010.jsp> accessed 02/10/11

Directorate of General Statistics and Economical Information (2006) *Recensement agricole de mai 2005* (in French) available on request from <http://statbel.fgov.be/en/statistics/figures/>

*Honeybees*
in Potts S.G., Roberts S.P.M., Dean R., Marris G., Brown M.A., Jones R., Neumann P., and Settele J. (2010b) Declines of Managed Honeybees and Beekeepers in Europe; *Journal of Apicultural Research 49*, 15-22

European Commission (2010) *Commission Regulation (EU) No 726/2010* <http://eur-lex.europa.eu/LexUriServ/LexUriServ.do?uri=OJ:L:2010:213:0029:0030:EN:PDF> accessed 11/11/13

***Bosnia and Herzegovina****Honeybees*Bosnia and Herzegovina Agency for Statistics (2006) *Number of livestock and livestock production in 2005* <http://www.bhas.ba/saopstenja/stocarstvo_2005.pdf> accessed 11/11/13

Bosnia and Herzegovina Agency for Statistics (2011) *Number of livestock and livestock production in 2010* <http://www.bhas.ba/saopstenja/2011/AGR_2011_004_01_BH.pdf> accessed 11/11/13

*Crops*
Bosnia and Herzegovina Agency for Statistics (2006) *Area harvested and production by crops 2005* <http://www.bhas.ba/saopstenja/Agriculture_2005.pdf> accessed 11/11/13

Bosnia and Herzegovina Agency for Statistics (2006) *Production of fruit and grapes in 2005* <http://www.bhas.ba/saopstenja/voce_2005.pdf> accessed 11/11/13

Bosnia and Herzegovina Agency for Statistics (2011) *Production of fruit and grapes in 2010* <http://www.bhas.ba/saopstenja/AGR_2010_001_01_BH.pdf> accessed 11/11/13

Bosnia and Herzegovina Agency for Statistics (2011) *Area harvested and production by crops 2010* <http://www.bhas.ba/saopstenja/AGR_2010_003_01-BH.pdf> accessed 11/11/13

FAOStat (2013) *Crops* <http://faostat.fao.org/site/567/DesktopDefault.aspx?PageID=567#ancor> accessed 11/11/13, last updated 08/08/13

***Bulgaria*** *Honeybees and Crops*
Ministry of Agriculture and Food (2007) *Agrarian Report 2007* <http://www.mzh.government.bg/MZH/Libraries/AgryReports/Annual_Report_2007.sflb.ashx> (in Bulgarian) accessed 11/11/13

Ministry of Agriculture and Food (2009) *Agrarian Report 2009* <http://www.mzh.government.bg/MZH/Libraries/AgryReports/agrarian_report_2009_EN.sflb.ashx> accessed 11/11/13

Ministry of Agriculture and Food (2011) *Agrarian Report 2011* <http://www.mzh.government.bg/MZH/Libraries/AgryReports/AD_2011_final_annexes_en.sflb.ashx> accessed 11/11/13

European Commission (2010) *Commission Regulation (EU) No 726/2010* <http://eur-lex.europa.eu/LexUriServ/LexUriServ.do?uri=OJ:L:2010:213:0029:0030:EN:PDF> accessed 11/11/13

***Croatia*** *Crops and Honeybees*
Croatian Bureau of Statistics (2010) *Statistical yearbook 2010* <http://www.dzs.hr/Hrv_Eng/ljetopis/2010/SLJH2010.pdf> accessed 11/11/13

Croatian Bureau of Statistics (2011) *Statistical Yearbook of the Republic of Croatia 2011* <http://www.dzs.hr/Hrv_Eng/ljetopis/2011/SLJH2011.pdf> accessed 11/11/13

***Cyprus*** *Crops*
Department of Agriculture of Cyprus (2005) *Agricultural statistics 2005*, Series ii, Report No.37, Republic of Cyprus

Republic of Cyprus Statistical Service (2012) *Agricultural Statistics 2009-2010*; <http://www.cystat.gov.cy/mof/cystat/statistics.nsf/All/F9A639CC1FAC3F3FC2257777004049A9/$file/AGRI_STAT-2009_2010-171212.pdf?OpenElement> accessed 11/11/13

*Honeybees*
Department of agriculture (2012) Data available on request

European Commission (2010) *Commission Regulation (EU) No 726/2010* <http://eur-lex.europa.eu/LexUriServ/LexUriServ.do?uri=OJ:L:2010:213:0029:0030:EN:PDF> accessed 11/11/13

***Czech Republic*** *Crops*
Czech Statistical office (2006) *Final Figures on Farm Crops Harvest 2005* <http://www.czso.cz/csu/2006edicniplan.nsf/engpubl/2102-06-in_2005> accessed 11/11/13

Czech Statistical office (2011) *Final Figures on Farm Crops Harvest 2010* <http://www.czso.cz/csu/2011edicniplan.nsf/engpubl/2102-11-eng_r_2011> accessed 11/11/13

FAOStat (2013) *Crops* <http://faostat.fao.org/site/567/DesktopDefault.aspx?PageID=567#ancor> accessed 11/11/13, last updated 08/08/13

*Honeybees*
Czech Statistical Office (2012) *Statistical Yearbook of the Czech Republic 2012* (Table 13.25 Apiculture) <http://www.czso.cz/csu/2012edicniplan.nsf/engkapitola/0001-12-eng_r_2012-1300> accessed 11/11/13

***Denmark*** *Crops*
Danish Plant Directorate (2012) *Planted Crop Area 2005 and 2010*

*Honeybees*in Potts S.G., Roberts S.P.M., Dean R., Marris G., Brown M.A., Jones R., Neumann P. and Settele J. (2010b) Declines of Managed Honeybees and Beekeepers in Europe; *Journal of Apicultural Research 49*, 15-22

European Commission (2010) *Commission Regulation (EU) No 726/2010* <http://eur-lex.europa.eu/LexUriServ/LexUriServ.do?uri=OJ:L:2010:213:0029:0030:EN:PDF> accessed 11/11/13

***Estonia*** *Crops*
Estonian agricultural registers and information board (2011) *PRIA-st toetuse saamiseks taotletud pindalad põllukultuuride kaupa 2005*

Statistics Estonia (2012) *AG033 Harvested Area of Field Crops* <http://pub.stat.ee/px-web.2001/I_Databas/Economy/01Agriculture/02Agricultural_production/02Crop_production/02Crop_production.asp> accessed 11/11/13, last updated 29/07/13

Statistics Estonia (2012) *AG060: Orchards and Berry Plantations by county* <http://pub.stat.ee/px-web.2001/I_Databas/Economy/01Agriculture/02Agricultural_production/02Crop_production/02Crop_production.asp> accessed 11/11/13, last updated: 29/07/13

*Honeybees*
Statistics Estonia (2011) *AG29: Supply Balance of Honey* <http://pub.stat.ee/px-web.2001/dialog/varval.asp?ma=AG29&ti=SUPPLY+BALANCE+OF+HONEY&path=../I_databas/Economy/01Agriculture/02Agricultural_production/06Supply_balance_sheets_of_agricultural_products/&search=AG29%3A+SUPPLY+BALANCE+OF+HONEY&lang=1> accessed 11/11/13, last updated 15/02/12

***Finland*** *Crops*
Tike (2006) *Production of the Main Crops* 2005 <http://www.maataloustilastot.fi/sites/default/files/viljelykasvien_sato_2005.pdf> accessed 11/11/13

Tike (2006) *Utilised Agricultural Area 2005* <http://www.maataloustilastot.fi/sites/default/files/kaytossa_oleva_maatalousmaa_2005.pdf> accessed 11/11/13

Tike (2011) *Production of the Main Crops* 2010 <http://www.maataloustilastot.fi/sites/default/modules/pubdlcnt/pubdlcnt.php?file=http://www.maataloustilastot.fi/sites/default/files/viljelykasvien_sato_2010_0.xls&nid=1763> accessed 11/11/13

Tike (2006) *Horticultural Statistics 2005* <http://www.maataloustilastot.fi/en/horticultural-statistics-2005_en> accessed 11/11/13, last updated 31/03/06

Tike (2011) *Horticultural Statistics 2010* <http://www.maataloustilastot.fi/en/horticultural-statistics-2010_en> accessed 11/11/13 last updated 31/03/11

*Honeybees*in Potts S.G., Roberts S.P.M., Dean R., Marris G., Brown M.A., Jones R., Neumann P. and Settele J. (2010b) Declines of Managed Honeybees and Beekeepers in Europe; *Journal of Apicultural Research 49*, 15-22

European Commission (2010) *Commission Regulation (EU) No 726/2010* <http://eur-lex.europa.eu/LexUriServ/LexUriServ.do?uri=OJ:L:2010:213:0029:0030:EN:PDF> accessed 11/11/13

***France****Crops*
Area of the main crops in mainland France (includes Corsica island) (2011) <http://agreste.maapar.lbn.fr/ReportFolders/ReportFolders.aspx?CS_referer=&CS_ChosenLang=fr> accessed 02/10/11, now archived at <http://archive.is/gYL1> accessed 11/11/13

*Honeybees*
FranceAgriMer (2012) *Audit économique de la filière apicole française*

<http://www.franceagrimer.fr/content/download/17875/141072/file/Audit_de_la_filili%E8re_apicole_2012.pdf> accessed 11/11/13

***Germany*** *Honeybees*in Potts S.G., Roberts S.P.M., Dean R., Marris G., Brown M.A., Jones R., Neumann P., and Settele J. (2010b) Declines of Managed Honeybees and Beekeepers in Europe; *Journal of Apicultural Research 49*, 15-22

European Commission (2010) *Commission Regulation (EU) No 726/2010* <http://eur-lex.europa.eu/LexUriServ/LexUriServ.do?uri=OJ:L:2010:213:0029:0030:EN:PDF> accessed 11/11/13

*Crops*FAOStat (2013) *Crops* <http://faostat.fao.org/site/567/DesktopDefault.aspx?PageID=567#ancor> accessed 11/11/13, last updated 08/08/13

Statistisches Bundesamt (2011) *Landwirtschaftliche Bodennutzung und pflanzliche Erzeugung - Fachserie 3 Reihe 3 – 2010* <https://www.destatis.de/DE/Publikationen/Thematisch/LandForstwirtschaft/Bodennutzung/BodennutzungErzeugung.html> (In German) accessed 11/11/13

Statistisches Jarbuch (2011) *Landwirtschaft und Forsten. Wirtschaftsverlag NW GmbH Bremerhaven* <http://www.bmelv-statistik.de/de/statistisches-jahrbuch/kap-c-landwirtschaft/> (in German) accessed 11/11/13

***Georgia*** *Crops*
Ministry of Economic Development of Georgia (2008) *Agriculture of Georgia 2007*, Ministry of Economic Development of Georgia, Tbilisi: Available on request from [www.geostat.ge](http://www.geostat.ge)

FAOStat (2013) *Crops* <http://faostat.fao.org/site/567/DesktopDefault.aspx?PageID=567#ancor> accessed 11/11/13, last updated 08/08/13

*Honeybees*
Ministry of Economic Development of Georgia (2011) *Honeybee numbers* Data available on request from [www.geostat.ge](http://www.geostat.ge)

***Greece****Crops and Honeybees*
Hellenic Statistics Authority (2011) *Agricultural Statistics of Greece 2010* available on request from <http://www.statistics.gr/>

Hellenic Statistics Authority (2011) *Honeybee numbers* available on request from <http://www.statistics.gr/>

Hellenic Statistics Authority (2005) *Agricultural Statistics of Greece 2005* <http://dlib.statistics.gr/Book/GRESYE_02_0903_00075.pdf> accessed 11/11/13

European Commission (2010) *Commission Regulation (EU) No 726/2010* <http://eur-lex.europa.eu/LexUriServ/LexUriServ.do?uri=OJ:L:2010:213:0029:0030:EN:PDF> accessed 11/11/13

***Hungary****Crops*
Hungarian Central Statistical Office (2011) *Production of main field crops, vegetables, grass and reed*; <http://statinfo.ksh.hu/Statinfo/themeSelector.jsp?page=2&szst=OMN> accessed 11/11/13

Hungarian Central Statistical Office (2006) *Farm Structure Survey 2005 - Distribution of orchard area and harvested production by fruit and berry species, 2005* <http://portal.ksh.hu/pls/ksh/docs/eng/agrar/html/tabl2_18_05.html> accessed 11/11/13

Hungarian Central Statistical Office (2011) *Use of land area by land-use categories and by legal forms, 31 May (1990–)* <http://portal.ksh.hu/pls/ksh/docs/eng/xstadat/xstadat_annual/i_omf001a.html> accessed 11/11/13

Hungarian Central Statistical Office (2012) *Production and use of main fruits (2009–)* <http://statinfo.ksh.hu/Statinfo/themeSelector.jsp?page=2&szst=OMN> accessed 11/11/13

Hungarian Central Statistical Office (2012) *Production and use of main crops grown on arable land (2009–)* <http://www.ksh.hu/docs/eng/xstadat/xstadat_annual/i_omn002d.html> accessed 11/11/13

*Honeybees*
Institute of Animal Science and Biodiversity (2011) *Number of hives (honey-bee colonies) in Hungary*, Data available on request

Hungarian National Apiary Association (2011) Data available on request

***Italy****Crops*
National Institute of Statistics of Italy (2011) *Total Area and Production* <http://dati.istat.it/Index.aspx?lang=en> accessed 11/11/13

*Honeybees*in Potts S.G., Roberts S.P.M., Dean R., Marris G., Brown M.A., Jones R., Neumann P., and Settele J. (2010b) Declines of Managed Honeybees and Beekeepers in Europe; *Journal of Apicultural Research 49*, 15-22

European Commission (2010) *Commission Regulation (EU) No 726/2010* <http://eur-lex.europa.eu/LexUriServ/LexUriServ.do?uri=OJ:L:2010:213:0029:0030:EN:PDF> accessed 11/11/13

***Ireland*** *Crops*
Central Statistical Office of Ireland (2011) *AQA01: Area Farmed in June (1991-2007) by Region, Type of Land Use and Year* <http://www.cso.ie/px/pxeirestat/Statire/SelectVarVal/Define.asp?maintable=AQA01&PLanguage=0> accessed 11/11/13

Department of Agriculture Fisheries and Food (2011) Data available on request <http://www.agriculture.gov.ie/>

FAOStat (2013) *Crops* <http://faostat.fao.org/site/567/DesktopDefault.aspx?PageID=567#ancor> accessed 11/11/13, last updated 08/08/13

*Honeybees*in Potts S.G., Roberts S.P.M., Dean R., Marris G., Brown M.A., Jones R., Neumann P., and Settele J. (2010b) Declines of Managed Honeybees and Beekeepers in Europe; *Journal of Apicultural Research 49*, 15-22

European Commission (2010) *Commission Regulation (EU) No 726/2010* <http://eur-lex.europa.eu/LexUriServ/LexUriServ.do?uri=OJ:L:2010:213:0029:0030:EN:PDF> accessed 11/11/13

***Latvia*** *Crops*Central Statistics Bureau (2011) *LAG015 Sown Area of Agricultural Crops* <http://data.csb.gov.lv/Selection.aspx?px_tableid=lauks\Ikgad%C4%93jie+statistikas+dati\03Augk\LA0150.px&px_language=en&px_type=PX&px_db=lauks&rxid=7d07f77a-cba9-48f0-91d2-ac5cca6c24e2> accessed11/11/13, last updated 10/10/13

Ministry of Agriculture Republic of Latvia (2006) *Agriculture and Rural Area of Latvia 2006*; <http://www.zm.gov.lv/doc_upl/ZM_Gada_parskats_LOW%282%29.pdf> accessed 11/11/13

Ministry of Agriculture Republic of Latvia (2011) *Agriculture and Rural Area of Latvia 2011*; <http://www.zm.gov.lv/doc_upl/LS_gada_zinojums_2011.pdf> (in Latvian) accessed 11/11/13

*Honeybees*Central Statistics Bureau (2011) – Data available on request

European Commission (2010) *Commission Regulation (EU) No 726/2010* <http://eur-lex.europa.eu/LexUriServ/LexUriServ.do?uri=OJ:L:2010:213:0029:0030:EN:PDF> accessed 11/11/13

***Lithuania*** *Crops*
Statistics Lithuania (2012*) M5010305: Orchards and berry plantations (since 2003) by kind, type of farm* <http://db1.stat.gov.lt/statbank/SelectVarVal/Define.asp?MainTable=M5010305&PLanguage=1&PXSId=0&ShowNews=OFF> accessed 11/11/13, last updated 10/07/13

Statistics Lithuania (2012) *M5010304:* *Field vegetables by kind, type of farm* <http://db1.stat.gov.lt/statbank/SelectVarVal/Define.asp?MainTable=M5010304&PLanguage=1&PXSId=0&ShowNews=OFF> accessed 11/11/13, last updated 26/02/13

Statistics Lithuania (2012) *M5010302: Farm crops in the country by kind, type of farm* <http://db1.stat.gov.lt/statbank/SelectVarVal/Define.asp?MainTable=M5010302&PLanguage=1&PXSId=0&ShowNews=OFF> accessed 11/11/13, last updated 10/10/13

*Honeybees*
Statistica Lithuania (2013) *M5010402:* *Number of livestock, poultry, rabbits and beehives by administrative territory, kind*; <http://db1.stat.gov.lt/statbank/SelectVarVal/Define.asp?MainTable=M5010402&PLanguage=1&PXSId=0&ShowNews=OFF> accessed 11/11/13, last updated 21/05/13

***Luxembourg*** *Crops*
ASTA (Administration des Services Techniques de l’Agriculture) (2011) *Fruit tree area in Luxembourg* Available on request from Service d'Economie Rurale

Service d'economie Rurale (2011) *Production of crops from arable land and fodder production* <http://www.ser.public.lu/statistics/crop_production/mengen_marktfruchtbau.pdf> accessed 11/11/13

*Honeybees*in Potts S.G., Roberts S.P.M., Dean R., Marris G., Brown M.A., Jones R., Neumann P., and Settele J. (2010b) Declines of Managed Honeybees and Beekeepers in Europe; *Journal of Apicultural Research 49*, 15-22

European Commission (2010) *Commission Regulation (EU) No 726/2010* <http://eur-lex.europa.eu/LexUriServ/LexUriServ.do?uri=OJ:L:2010:213:0029:0030:EN:PDF> accessed 11/11/13

***Macedonia (The Former Yugoslav Republic of)*** *Crops and Honeybees*
Republic of Macedonia State Statistical Office (2010) *Statistical Yearbook of the Republic of Macedonia 2010 – Chapter 10: Agriculture* <http://makstat.stat.gov.mk/Publikacii/SG2010/SG2010.zip> accessed 11/11/13

*Crops*FAOStat (2013) *Crops* <http://faostat.fao.org/site/567/DesktopDefault.aspx?PageID=567#ancor> accessed 11/11/13, last updated 08/08/13

***Malta*** *Crops*FAOStat (2013) *Crops* <http://faostat.fao.org/site/567/DesktopDefault.aspx?PageID=567#ancor> accessed 11/11/13, last updated 08/08/13

*Honeybees*
National Statistics Office Malta (2004) *Apiculture Census* <http://www.nso.gov.mt/statdoc/document_file.aspx?id=136> accessed 11/11/13

European Commission (2010) *Commission Regulation (EU) No 726/2010* <http://eur-lex.europa.eu/LexUriServ/LexUriServ.do?uri=OJ:L:2010:213:0029:0030:EN:PDF> accessed 11/11/13

***Moldova*** *Crops*
National Bureau of Statistics of the Republic of Moldova (2011) *Sown areas, production and average yield by agricultural crops, categories of producers, 1980-2010* <http://statbank.statistica.md/pxweb/Dialog/varval.asp?ma=AGR0201_en&ti=Sown+areas%2C+production+and+average+yield+by+agricultural+crops%2C+categories+of++producers%2C+1980-2010&path=../Database/EN/16%20AGR/AGR02/&lang=3> accessed 11/11/13, last updated 26/10/12

National Bureau of Statistics of the Republic of Moldova (2011) *Perennial plantations by plants and categories of producers, 1980-2010* <http://statbank.statistica.md/pxweb/Dialog/varval.asp?ma=AGR0202_en&ti=Perennial+plantations+by+plants+and+categories+of+producers%2C+1980-2010&path=../Database/EN/16%20AGR/AGR02/&lang=3> accessed 11/11/13, last updated 26/10/12

FAOStat (2013) *Crops* <http://faostat.fao.org/site/567/DesktopDefault.aspx?PageID=567#ancor> accessed 11/11/13, last updated 08/08/13

*Honeybees*National Bureau of Statistics of the Republic of Moldova (2011) *Livestock as January 1 by categories of producers, 1980-2011* <http://statbank.statistica.md/pxweb/Dialog/varval.asp?ma=AGR0301_en&ti=Livestock+as+January+1+by+categories+of+producers%2C+1980-2011&path=../Database/EN/16%20AGR/AGR03/&lang=3> accessed 11/11/13, updated 05/11/12

***Montenegro*** *Crops*
Statistical office of Montenegro (2012) *Fruit trees and production of fruit* <http://www.monstat.org/eng/page.php?id=276&pageid=62> accessed 11/11/13

Statistical office of Montenegro (2012) *Production of Vegetable Crops* <http://www.monstat.org/eng/page.php?id=276&pageid=62> accessed 11/11/13

Statistical office of Montenegro (2011) *Area of small fruit production* Data available on request from [www.monstat.org](http://www.monstat.org)

*Honeybees*
Statistical Office of Montenegro (2011) *The number cattle, poultry and beehives* <http://www.monstat.org/userfiles/file/stocna/stocna%20eng/number%20cattle,poultry.xls> accessed 11/11/13

***Netherlands****Crops*
Centraal Bureau voor de Statistiek (CBS) (2011) *Area of outdoor crops in the Netherlands*; StatLine databank

*Honeybees*
in Potts S.G., Roberts S.P.M., Dean R., Marris G., Brown M.A., Jones R., Neumann P., and Settele J. (2010b) Declines of Managed Honeybees and Beekeepers in Europe; *Journal of Apicultural Research 49*, 15-22

European Commission (2010) *Commission Regulation (EU) No 726/2010* <http://eur-lex.europa.eu/LexUriServ/LexUriServ.do?uri=OJ:L:2010:213:0029:0030:EN:PDF> accessed 11/11/13

***Norway****Crops*
Statistics Norway (2011) *Table 06046: Yields of Various Horticultural Crops* <http://statbank.ssb.no/statistikkbanken/Default_FR.asp?Productid=10.04&PXSid=0&nvl=true&PLanguage=1&tilside=selecttable/MenuSelP.asp&SubjectCode=10>

Statistics Norway (2011) *Table 04415: Agricultural Area per 31 July, by crop* <https://www.ssb.no/statistikkbanken/selectvarval/Define.asp?subjectcode=&ProductId=&MainTable=JordArealDekar&nvl=&PLanguage=1&nyTmpVar=true&CMSSubjectArea=jord-skog-jakt-og-fiskeri&KortNavnWeb=jordbruksareal&StatVariant=&checked=true> accessed 11/11/13, last updated 26/11/12

*Honeybees*
in Potts S.G., Roberts S.P.M., Dean R., Marris G., Brown M.A., Jones R., Neumann P., and Settele J. (2010b) Declines of Managed Honeybees and Beekeepers in Europe; *Journal of Apicultural Research 49*, 15-22

***Poland****Crops*
Central Statistical Office (2006) *Wyniki produkcji roslinnej w 2005 r*: <http://www.stat.gov.pl/gus/5840_1589_PLK_HTML.htm> (in Polish) accessed 11/11/13

Central Statistical Office (2011) *Wyniki produkcji roslinnej w 2010 r*: <http://www.stat.gov.pl/gus/5840_1589_PLK_HTML.htm> (in Polish) accessed 11/11/13

Central Statistical Office (2012) *Statistical Yearbook of Agriculture 2011*: <http://www.stat.gov.pl/cps/rde/xbcr/gus/sy_statistical_yearbook_agriculture_2011.pdf> accessed 11/11/13

*Honeybees*
in Potts S.G., Roberts S.P.M., Dean R., Marris G., Brown M.A., Jones R., Neumann P., and Settele J. (2010b) Declines of Managed Honeybees and Beekeepers in Europe; *Journal of Apicultural Research 49*, 15-22

European Commission (2010) *Commission Regulation (EU) No 726/2010* <http://eur-lex.europa.eu/LexUriServ/LexUriServ.do?uri=OJ:L:2010:213:0029:0030:EN:PDF> accessed 11/11/13

***Portugal****Crops*
Statistics Portugal (2012) *Main crops surface (ha) by Geographic localization (NUTS - 2002) and Specie; Annual* [*http://www.ine.pt/xportal/xmain?xpid=INE&xpgid=ine_indicadores&indOcorrCod=0000018&contexto=bd&selTab=tab2*](http://www.ine.pt/xportal/xmain?xpid=INE&xpgid=ine_indicadores&indOcorrCod=0000018&contexto=bd&selTab=tab2) accessed 11/11/13, last updated 07/06/13

FAOStat (2013) *Crops* <http://faostat.fao.org/site/567/DesktopDefault.aspx?PageID=567#ancor> accessed 11/11/13 last updated 08/08/13

*Honeybees*
in Potts S.G., Roberts S.P.M., Dean R., Marris G., Brown M.A., Jones R., Neumann P., and Settele J. (2010b) Declines of Managed Honeybees and Beekeepers in Europe; *Journal of Apicultural Research 49*, 15-22

European Commission (2010) *Commission Regulation (EU) No 726/2010* <http://eur-lex.europa.eu/LexUriServ/LexUriServ.do?uri=OJ:L:2010:213:0029:0030:EN:PDF> accessed 11/11/13

***Romania****Crops and Honeybees*
National Institute of Statistics (2011) *Statistical yearbook 2010, Table 14.10 Cultivated Area by Main crops and Table 14.15 Livestock* <http://www.insse.ro/cms/files/Anuar%20statistic/14/14%20Agricultura%20si%20silvicultura_en.pdf> accessed 11/11/13

FAOStat (2013) *Crops* <http://faostat.fao.org/site/567/DesktopDefault.aspx?PageID=567#ancor> accessed 11/11/13, last updated 08/08/13

***Serbia****Honeybees*
Statistical Office of the Republic of Serbia (2011) *Number of Beehives* <http://webrzs.stat.gov.rs/WebSite/Public/ReportResultView.aspx?rptKey=indId%3d13020209IND01%262%3d%23Last%233%2635%3d6%2c7%2c8%26sAreaId%3d13020209%26dType%3dName%26lType%3dEnglish> accessed 11/11/13, last updated 01/2012

*Crops*
Statistical Office of the Republic of Serbia (2011) *Crop Farming* <http://webrzs.stat.gov.rs/WebSite/Public/ReportView.aspx?rptKey=indId%3d13020209IND01%262%3d%23Last%233%2635%3d6%2c7%2c8%26sAreaId%3d13020209%26dType%3dName%26lType%3dEnglish&b=1> accessed 11/11/13, last updated 01/2012

***Slovakia****Crops*
Statistical Office of the Slovak Republic (2011) *Area of fruit crops* Data available on request from <http://www.statistics.sk>

Statistical Office of the Slovak Republic (2011) *Yields of selected agricultural crops (1970 - 2010)* <http://www.statistics.sk/pls/elisw/objekt.send?uic=709&m_so=17> accessed 11/11/13

*Honeybees*
in Potts S.G., Roberts S.P.M., Dean R., Marris G., Brown M.A., Jones R., Neumann P., and Settele J. (2010b) Declines of Managed Honeybees and Beekeepers in Europe; *Journal of Apicultural Research 49*, 15-22

European Commission (2010) *Commission Regulation (EU) No 726/2010* <http://eur-lex.europa.eu/LexUriServ/LexUriServ.do?uri=OJ:L:2010:213:0029:0030:EN:PDF> accessed 11/11/13

***Slovenia****Crops*
Statistical office of the Republic of Slovenia (2013) *Production of crops (ha, t, t/ha), Slovenia, annually* <http://pxweb.stat.si/pxweb/Dialog/varval.asp?ma=1502402E&ti=&path=../Database/Environment/15_agriculture_fishing/04_crop_production/01_15024_crops_area/&lang=1> accessed 11/11/13, last updated 28/03/13

Statistical office of the Republic of Slovenia (2013) *Production of vegetables (ha, t, t/ha), Slovenia, annually* <http://pxweb.stat.si/pxweb/Dialog/varval.asp?ma=1502403E&ti=&path=../Database/Environment/15_agriculture_fishing/04_crop_production/01_15024_crops_area/&lang=1> accessed 11/11/13, last updated 28/03/13

Statistical Office of the Republic of Slovenia (2011) *Production of fruit in orchard plantations and olives in olive plantations (ha, number of trees, t, t/ha), Slovenia, annually* <http://pxweb.stat.si/pxweb/Dialog/varval.asp?ma=1502404E&ti=&path=../Database/Environment/15_agriculture_fishing/04_crop_production/01_15024_crops_area/&lang=1> accessed 11/11/13, last updated 28/03/13

*Honeybees*
Statistical office of the Republic of Slovenia (2011) *Honeybee numbers* Data available on request

***Spain****Crops*
MARM (2012) Statistical Yearbook 2011: *Chapter 13 Crop Areas and Production* <http://www.magrama.gob.es/es/estadistica/temas/estad-publicaciones/anuario-de-estadistica/2011/default.aspx> (in Spanish)

MARM (2006) *Statistical Yearbook 2006: Crop Areas and Production* <http://www.magrama.gob.es/es/estadistica/temas/publicaciones/anuario-de-estadistica/2011/default.aspx> (in Spanish) accessed 11/11/13

*Honeybees*in Potts S.G., Roberts S.P.M., Dean R., Marris G., Brown M.A., Jones R., Neumann P., and Settele J. (2010b) Declines of Managed Honeybees and Beekeepers in Europe; *Journal of Apicultural Research 49*, 15-22

European Commission (2010) *Commission Regulation (EU) No 726/2010* <http://eur-lex.europa.eu/LexUriServ/LexUriServ.do?uri=OJ:L:2010:213:0029:0030:EN:PDF> accessed 11/11/13

***Sweden****Crops*
Swedish board of agriculture (2011) *Jordbruksstatistisk årsbok, Svensk Raps AB*

FAOStat (2013) *Crops* <http://faostat.fao.org/site/567/DesktopDefault.aspx?PageID=567#ancor> accessed 11/11/13, last updated 08/08/13

*Honeybees*
Preben Kristiansen (Pers comm. 2011) CBR

***Switzerland*** *Crops*
Fedaral Statistics office (2012) *Flächen der Obstkulturen*; <http://www.bfs.admin.ch/bfs/portal/de/index/themen/07/03/blank/data/01/02.html> accessed 11/11/13, last updated 16/11/12 (in German)

Fedaral Statistics office (2012) *Landwirtschaftliche Betriebe nach Jahr, Kanton und Zone - Landwirtschaftliche Nutzfläche (LN) und Nutztiere auf Klassifizierungsebene 2* <http://www.pxweb.bfs.admin.ch/Dialog/varval.asp?ma=px-d-07-2A02&path=../Database/German_07%20-%20Land-%20und%20Forstwirtschaft/07.2%20-%20Landwirtschaft/&lang=1&prod=07&openChild=true&secprod=2> (in German) accessed 11/11/13, last updated 20/08/13

*Honeybees*Fedaral Statistics office (2012) *Landwirtschaftliche Betriebe nach Jahr, Kanton und Zone - Landwirtschaftliche Nutzfläche (LN) und Nutztiere auf Klassifizierungsebene 2* <http://www.pxweb.bfs.admin.ch/Dialog/varval.asp?ma=px-d-07-2A02&path=../Database/German_07%20-%20Land-%20und%20Forstwirtschaft/07.2%20-%20Landwirtschaft/&lang=1&prod=07&openChild=true&secprod=2> (in German) accessed 11/11/13, last updated 20/08/13

***Turkey*** *Crops*
Turkish Statistical institute (2011) *Crop Production Statistics* <http://www.turkstat.gov.tr/PreTablo.do?alt_id=1001> accessed 11/11/13

FAOStat (2013) *Crops* <http://faostat.fao.org/site/567/DesktopDefault.aspx?PageID=567#ancor> accessed 11/11/13, last updated 08/08/13

*Honeybees*
Turkish Statistical Institute (2011) *Livestock Statistics* <http://www.turkstat.gov.tr/PreTablo.do?alt_id=1002> accessed 11/11/13

***United Kingdom*** *Crops*
DEFRA (2012a) *Agriculture in the United Kingdom* <https://www.gov.uk/government/statistical-data-sets/agriculture-in-the-united-kingdom> accessed 11/11/13, last updated 25/07/12

DEFRA (2012b) *Basic Horticultural Statistics* <https://www.gov.uk/government/publications/basic-horticultural-statistics> accessed 11/11/13, last updated 31/07/2013

*Honeybees*in Potts S.G., Roberts S.P.M., Dean R., Marris G., Brown M.A., Jones R., Neumann P., and Settele J. (2010b) Declines of Managed Honeybees and Beekeepers in Europe; *Journal of Apicultural Research 49*, 15-22

***Ukraine*** *Crops and Honeybees*
State Statistics Committee of Ukraine (2012) *Agriculture of Ukraine 2011*
<http://www.ukrstat.gov.ua/druk/publicat/kat_u/2012/09_2012/zb_sg_2011.zip> accessed 11/11/13

*Country specific crop area data transformations, clarifications and assumptions*

For all countries and datasets only bearing or harvested area was included where this was distinct from total sown area. Unless otherwise stated, reported area of beans and nuts were assumed to be non-insect pollinated species only.

In several southern European countries, tree and small fruit crops are reported in trees or bushes rather than area. To convert these into hectares, average densities of trees/ha were estimated from the average number of trees per hectare reported by the Hellenic Statistic Agency [77] and Republic of Macedonia State Statistical Office [78]. For soft fruit bushes where no official density estimates were available, a conservative estimate of 1000 per hectare was used. This conversion was only used for Southern European nations and usually produced more conservative estimates than those used by the FAO.

**FAOStat (2012) data** Beans (Dry) were assumed to be synonymous with field beans (*Vicia faba*) due to widely observed relations between this figure and field beans in countries where both figures were available. Berries nes were taken as “other soft fruit” Fresh fruit nes and Stone fruit nes were taken as “other top fruit”.

**Albania** FAOstat (2012) was used for all crops due to very limited official data availability.

**Armenia** FAOstat (2012) was used for all crops due to very limited official data availability.

**Austria** All fruit crops include both summer and winter planted area and both intensive and extensive area where applicable (e.g Strawberries). For Cucumbers and Peppers, only outdoor planted area was included.

**Belarus** FAOstat (2012) was used for all crops except Rapeseed and Buckwheat due to limited official data availability

**Belgium** Field and dry beans were assumed to be *Vica faba* only. Only courgettes and strawberries grown outdoors were included within the honeybee pollinated crop area.

**Bosnia and Herzegovina** FAOstat (2012) was used for Strawberries, Raspberries in 2005 as these crops were not officially reported in 2005. Area of tree fruit crops were estimated from the number of trees.

**Bulgaria** Dried beans was assumed to pertain to field beans *Vicia faba*. Morello cherries are synonymous with sour cherries.

**Croatia** Dry beans were assumed to be synonymous with field beans *Vicia faba*.

**Czech Republic** FAOstat (2012) data was used for Broad/Field Beans which are not officially reported and tree fruit crops and currants which are officially reported in the number of trees/bushes.

**Denmark** Area of currants was counted as blackcurrants and area of Cherries and Plums was counted as Sweet Cherries, as these have lower recommended stocking rate assumptions than other similar crops. Area of other pulses was estimated as the total area of pulses minus the area of all named pulses.

**Estonia** Area of Other fruits and Berries was counted as other top fruits as these have lower recommended densities than “other soft fruit”. Area of Apples and Pears was taken as Apples following FAOStat (2012).

**France** Area of Blackcurrants and Blueberries was counted as blackcurrants as these have the lowest recommended density assumptions of the two crops. For strawberries in 2005, field production was estimated as the total area multiplied by the proportion of 2010 crop grown in the open.

**Georgia** FAOstat (2012) was used for all crops due to limited availability of official data. Data from the National Statistics Office of Georgia (2008) indicates that dry beans refers to haricot beans (*Passeolus vulgaris*) which are not pollinated by honeybees.

**Germany** FAOstat (2012) was used for tree fruit data and poppy seed in 2005 and for Apricots, Peaches, Soft Fruits (except strawberries) and poppy seed in 2010.

**Italy** Area of Green Beans and Kidney Beans was counted as honeybee pollinated as kidney beans are unlikely to occupy a large area based on FAOstat (2012). Only area of outdoor grown watermelon, strawberry and courgette were included. Quinces, Sesame seed and Pomegranate are not reported separately in 2010

**Ireland** FAOstat (2012) data was used for Broad/Field Beans which are not officially reported.

**Hungary** For soft fruits, the proportion of total production accounted for each reported species was used to estimate the proportion of their crop area. 2010 Currants were assumed to be blackcurrants. Peppers were included due to the high scale of production which is mostly field growth. FAOstat (2012) data was used for 2010 Almond, Chestnut, Quince, Gooseberry and Other Top fruit area which were not nationally reported for 2005.

**Malta** FAOstat (2012) was used for all crops as no national data is available. 2003 Honeybee numbers were used for 2005 as this was the most recently available data.

**Macedonia** Area of tree fruit crops were estimated from the number of trees. Field/Broad Bean area was taken as the total maincrop area of beans plus half the area of intercrop beans. FAOstat (2012) data was used for soft fruits and sesame seed as these are not officially available.

**Moldova** Melons and Gords was taken as cucurbits as these have a lower RSR. Area of “other tree fruits” was taken as the remainder of stone fruit and seed fruits after all named crop area had been accounted for. FAOStat (2012) was used for almond area.

**Montenegro** Area of tree fruit crops were estimated from the number of trees.

**Netherlands** The entry Red currants, Raspberries, Blackberries is counted as part other soft fruit in 2010. FAOStat (2012) estimates were used for Blueberry and Plum area in 2005 which were not recorded. No data was available for 2005 coverage of sour cherries.

**Norway** Oilseeds was taken as area of oilseed rape, approximately validated by FAOStat (2012). As no official 2010 data concerning the number of honeybee colonies could be obtained it was instead assumed that stocks had remained unchanged.

**Poland** Area of seed crops not accounted by grass seeds was assumed to be sainfoin. FAOStat (2012) estimates were used to disambiguate areas of “other” soft fruits and tree fruits which includes some crops which are not insect pollinated (Walnuts, Grapes and Hazelnuts).

**Portugal** FAOStat (2012) was used for strawberries, peaches & nectarines, avocados, broad beans and fruiting vegetables.

**Romania** FAOstat (2012) data was used for tree fruit crops and berries which are officially reported in the number of trees/bushes.

**Serbia** Area of tree fruit crops were estimated from the number of trees. Beans was synonymous with broad/field beans.

**Slovakia** FAOStat (2012) was used for field/broad beans. Gages are synonymous with plums.

**Slovenia** Berry bushes without strawberries was taken as “other soft fruit”. Oil turnip rape is synonymous with oilseed rape.

**Spain** Area of dried beans was taken as a total area of field/broad beans

**Sweden** FAOStat (2012) was used for Raspberries, blackcurrants, other berries, pears, cherries and plums in 2010 as this data is only officially collected every 3 years.

**Turkey** FAOStat (2012) was used for Cucumbers, Pumpkins and Melons. Area of tree fruit crops were estimated from the number of trees. Cornel was included as “other top fruit”.

**Ukraine** Area of “other top fruit” was estimated from the total area of seed and stone fruits minus any named crops. Area of “other soft fruit” was estimated from the total area of berries minus any named crops. FAOstat (2012) data was used for Broad/Field Beans.

**United Kingdom** Sweet cherries are not recorded separately in 2010 and are included in other top fruit. Honeybee numbers from 2000 are used for 2005 and values from 2008 are

**Supplemental Table 1.1** Regional classification of countries

| North | East | South | West |
| --- | --- | --- | --- |
| Denmark | Belarus | Albania | Austria |
| Estonia | Bulgaria | Armenia | Belgium |
| Finland | Czech Republic | Bosnia & Herzegovina | France |
| Ireland | Hungary | Croatia | Germany |
| Latvia | Moldova | Cyprus | Luxembourg |
| Lithuania | Poland | Georgia | Netherlands |
| Norway | Romania | Greece | Switzerland |
| Sweden | Slovakia | Italy |  |
| United Kingdom | Ukraine | Macedonia |  |
|  |  | Malta |  |
|  |  | Montenegro |  |
|  |  | Portugal |  |
|  |  | Serbia |  |
|  |  | Slovenia |  |
|  |  | Spain |  |
|  |  | Turkey |  |

**References**

1. Hellenic Statistics Authority *Agricultural Statistics of Greece 2005*; (2005) <http://dlib.statistics.gr/Book/GRESYE_02_0903_00075.pdf>
2. Republic of Macedonia State Statistical Office, *Census of Agriculture 2007*; (2007) <http://www.stat.gov.mk/Publikacii/PopisNaZemjodelstvo2007/BookI.pdf>
