# Supplementary material for: Agricultural Policies Exacerbate Honeybee Pollination Service Supply-Demand Mismatches Across Europe
Source: PLoS One. 2014 Jan 8;9(1):e82996. doi: 10.1371/journal.pone.0082996 (PMC3885438; doi:10.1371/journal.pone.0082996)
Supplement: Supporting Information S2 — Recommended Stocking Rates (RSR) of honeybee colonies per hectare of crops. (DOCX) [file pone.0082996.s002.docx]

**Supplemental S2 – Recommended Stocking Rates (RSR) of honeybee colonies per hectare of crops**

RSR values were collected from the cited sources. Where crop specific estimates were not available, a closely related crop was used as a proxy. If no closely related crop was available, then the average values of similar crops or those with similar floral morphology were used. Groundnuts/Peanuts (*Arachis hypogea*) were excluded since they show little to no benefit from insect pollination services and honeybees do not visit the flowers [79].

**Supplemental Table 1** – Summary of Recommended Stocking Rates

| Crop Name | Crop Species | RSR (L) | RSR (A) | RSR (U) | References |
| --- | --- | --- | --- | --- | --- |
| Alfalfa Seed | *Medicago sativa* | 2.5 | 7.9 | 14.8 | 18 |
| Almond | *Amygdalus communis* | 2.5 | 6 | 10 | 18; 80 |
| Apple | *Malus domestica* | 1 | 3.6 | 12.5 | 18; 80^a^ |
| Apricot | *Prunus armeniaca* | 3 | 4 | 5 | 80; 81 |
| Avocado | *Persea americana* | 2 | 4.5 | 7.5 | 18 |
| Blackberry | *Rubus fruticosus* | 2.5 | 6.7 | 10 | 18 |
| Blackcurrant^b^ | *Ribes nigrum* | 3 | 4.5 | 8 | 39 |
| Blueberry^c^ | *Vaccinium* spp. | 1.2 | 7.5 | 25 | 18 |
| Borage | *Borago officinalis* | 2 | 2 | 2 | 82 |
| Broad/Field Beans | *Vicia faba* | 2.5^d^ | 3.8 | 5 | 39 |
| Buckwheat | *Fagopyrum esculentum* | 2 | 3.5 | 5 | 83 |
| Carrot Seed^e^ | *Daucus carota* | 5 | 7.5 | 10 | 18 |
| Chestnut | *Castanea sativa* | 1.5 | 1.5 | 1.5 | 80 |
| Clover seed^f^ | *Trifolium* spp. | 2 | 5.8 | 15.7 | 18 |
| Cotton seed | *Gossypium hirsutum* | 0.5 | 5 | 12.4 | 18 |
| Cranberry | *Vaccinium oxycoccus* | 0.5 | 7.6 | 25 | 18 |
| Cucumber^g^ | *Cucumis sativus* | 0.3 | 5.5 | 10 | 18 |
| Cucurbits^h^ | *Cucurbita* spp. | 0.1 | 3.8 | 8 | 18 |
| Kiwifruit | *Actinidia deliciosa, A. chinensis* | 8 | 8 | 8 | 18 |
| Medlar^i^ | *Mespilus germanica* | 1 | 2.7 | 6.2 | Average |
| Melon/Cantaloupe | *Cucumis melo* | 0.5 | 4.4 | 12.4 | 18 |
| Oilseed Rape | *Brassica napus* | 1 | 2.8 | 5 | 18; 36; 37 |
| Other Mass Flowering^j^ | Various | 1.7 | 3.5 | 7.3 | Average |
| Other Nut^k^ | Various | 2 | 4.2 | 5.8 | Average |
| Other Soft Fruit^l^ | Various | 1.7 | 5.8 | 14.2 | Average |
| Other top fruit^m^ | Various | 1.3 | 3.3 | 5.7 | Average |
| Peach/Nectarine | *Prunus persica* | 0.2 | 1.8 | 2.5 | 18; 80 |
| Pear^n^ | *Pyrus communis* | 1 | 3.4 | 5 | 18; 80 |
| Plum^o^ | *Prunus domestica* | 2 | 3.2 | 5 | 18; 80 |
| Quince | *Cydonia oblonga* | 1 | 1 | 1 | 78 |
| Raspberry | *Rubus idaeus* | 0.5 | 2 | 2.5 | 18 |
| Redcurrant | *Ribes rubrum* | 4 | 4 | 4 | 39 |
| Runner/French Bean^p^ | *Phaseolus lunatus, P. vulgaris* | 1 | 2 | 2.5 | 84 |
| Sour Cherry | *Prunus cerasus* | 0 | 4.1 | 8 | 18 |
| Soybean | *Glycine max* | 1.4 | 1.4 | 1.4 | 18 |
| Strawberry | *Fragaria x ananassa* | 1.2 | 8.6 | 25 | 18 |
| Sunflower | *Helianthus annuus* | 1 | 2.1 | 4 | 18 |
| Sweet Cherry | *Prunus avium* | 1.3 | 4.2 | 5 | 18 |
| Vetch | *Vicia* spp. | 0.5 | 0.7 | 0.8 | 85 |
| Watermelon | *Citrullus lanatus* | 0.5 | 4.5 | 12.4 | 18 |

a: The lowest RSR cited in 5 is 0.6/ha, itself cited in 33. However, no study citing this value is under 80 years old when production systems were significantly less effective than present, resulting in trees producing less flowers. This value may still be appropriate for small holdings or traditional orchards (but see 30). The omission of this value does not significantly affect average RSR;
b: Used as a proxy for Gooseberry (*Ribes grossularia*);
c: Also Bilberries (*Vaccinium myrtillus*), these values include highbush, lowbush and rabbiteye blueberries;
d: 5 cites a value of 2.5 for Lima bean which has similar floral morphology and pollination requirements
e: Used as a proxy for Caraway (*Carum carvi*);
f: Average of all clover seed recommended densities listed in Delaplane and Mayer (2000);
g: Used as a Proxy for bell peppers and chilli peppers (*Capsicum annuum*) ;
h: Courgette, Pumpkin, Squash, Gourd and Pumpkin Seed;
i: Average values of other pome fruit – Apple, Pear and Quince ;
j: Sesame (*Sesamum indicum*), Sainfoin (*Onobrychis viciifolia*), Poppy (*Papaver* spp.), Mustard Seed (*Brassica* spp. & *Sinapis* spp.) and unspecified oilseeds. Average of Oilseed rape, Borage and Buckwheat;
k: Unspecified nut crops. Average of Chestnut and Almond;
l: Sea Buckthorn (*Hippophae* spp.), Elderberries (*Sambucus* spp.), Mullberries (*Morus* spp.), Chokeberries (*Aronia* spp.) and unspecified soft fruits. Average of stocking rates for Strawberry, Raspberry, Blackberry, Redcurrant, Blackcurrant, Blueberry and Cranberry;
m: Carob (*Ceratonia siliqua*), Persimmon (*Diospyros* spp.), Pomegranate (*Punica granatum*), Cornel (*Cornus mas*), Jujube (*Zizyphus jujube*), Feijoa (*Feijoa Sellowiana*) and unspecified fruit trees. Average of Apple, Avocado, Apricot, Pear, Peach, Plum, Quince, Sour Cherry and Sweet Cherry;
n: Includes Nashi (*Pyrus pyrifolia*);
o: Includes Gage;
p: Used as a proxy for other, unspecified pulse crops.

**References**

1. Blanche J.R., Hughes M., Ludwig J.A., Cunningham S.A. (2006), Do flower-tripping bees enhance yields in peanut varieties grown in north Queensland?; *Aust. J. Exp. Agr.* ***46***, 1529-1534.
2. Benedek, P. (2003) Insect pollination of temperate zone entomophilous fruit tree species and cultivar features affecting bee pollination in Kozma P., Nyeki J., Stolesz M. and Szabo Z. eds *Floral Biology, Pollination and Fertilisation in Temperate Zone Fruit and Grape*, 531–582, Akademiai Kiado, Budapest.
3. Austin P.T., Hewett E.W., Noiton D.A., Plummer J.A. (1996) Cross pollination of ‘Sundrop’ apricot (Prunus armeniaca L.) by honeybees; *New Zeal. J. Crop Hort.* ***24****,* 287-294.
4. El Hafid R., Blade S.F., Hoyano Y. (2002) *Trends in new crops and new uses* In: Janick J. Whipkey A. eds. (ASHS Press, Alexandria) Borage culture on the black soil zone of Alberta, Canada. 497–500.
5. Goodman R. Hepworth G., Kaczynski P., McKee B., Clarke S. and Bluett C. (2001) Honeybee pollination of buckwheat (*Fagopyrum esculentum* *Moench*) cv. Manor; *Aust. J. Exp. Agr.* ***41****,* 1217-1221.
6. Breeze T.D., Bailey A.P., Balcombe K.G. and Potts S.G. (2011) Pollination services in the UK: how important are honeybees?; *Agr. Ecosyst. Environ.* ***142****,* 137-143.
7. McGregor, S. E. (1976) *Insect pollination of cultivated crop plants* (Washington (DC). US Department of Agriculture, Agricultural Handbook 496). <http://gears.tucson.ars.ag.gov/book/index.html>
