# Supplementary material for: Agricultural Policies Exacerbate Honeybee Pollination Service Supply-Demand Mismatches Across Europe
Source: PLoS One. 2014 Jan 8;9(1):e82996. doi: 10.1371/journal.pone.0082996 (PMC3885438; doi:10.1371/journal.pone.0082996)
Supplement: Supporting Information S3 — Full Analytical results. (DOCX) [file pone.0082996.s003.docx]

**Supplemental S3 – Full Analytical results**

**Table S3.1** – Results of Supply Density and Density of Demand correlation analysis

| Variables | Coefficient (*ρ*) | P value |
| --- | --- | --- |
| SD 2005 x DDL 2005 | 0.01 | 0.9745 |
| SD 2010 x DDL 2010 | 0.12 | 0.4563 |
| SD 2005 x DDA 2005 | 0.49 | 0.0013 |
| SD 2010 x DDA 2010 | 0.48 | 0.0014 |
| SD 2005 x DDU 2005 | 0.59 | <0.0001 |
| SD 2010 x DDU 2010 | 0.59 | <0.0001 |

Variables = variables for which correlation was assessed, Coefficient (*ρ*) = the Spearman’s rank correlation coefficient, P value =the significance of the coefficient. In all analyses n=41. SD = Supply Density, the number of colonies available per hectare of insect-pollinated crop in 2005 or 2010. DD = Density of Demand, (the mean number of colonies required per hectare of insect-pollinated crop, weighted by the area of each crop and divided by two to take into account the potential for hive movement within a year) at lower (L), average (A) and upper (U) recommended stocking rate assumptions.

**Table S3.2** – Results of correlation analysis between Supply and Demand density variables and pollination service capacity

| Variables | Coefficient (r) | P value | | |
| --- | --- | --- | --- | --- |
| SD 2005 x PSCL 2005 | 0.97 | | <0.0001 |  |
| SD 2010 x PSCL 2010 | 0.79 | | <0.0001 |  |
| SD 2005 x PSCA 2005 | 0.99 | | <0.0001 |  |
| SD 2010 x PSCA 2010 | 0.99 | | <0.0001 |  |
| SD 2005 x PSCU 2005 | 0.98 | | <0.0001 |  |
| SD 2010 x PSCU 2010 | 0.96 | | <0.0001 |  |
| DDL 2005 x PSCL 2005 | -0.15 | | 0.3436 |  |
| DDL 2010 x PSCL 2010 | 0.06 | | 0.7022 |  |
| DDA 2005 x PSCA 2005 | 0.36 | | 0.0217 |  |
| DDA 2010 x PSCA 2010 | 0.47 | | 0.0019 |  |
| DDU 2005 x PSCU 2005 | 0.28 | | 0.0715 |  |
| DDU 2010 x PSCU 2010 | 0.42 | | 0.0059 |  |

Variables = variables for which correlation was assessed, Coefficient (r) = the Pearson’s product moment correlation coefficient, P value =the significance of the coefficient. In all analyses n=41. SD = Supply Density, the number of colonies available per hectare of insect-pollinated crop in 2005 or 2010. PSC = Pollination service capacity, the capacity of honeybee stocks to supply pollination services under lower (L), average (A) and upper (U) recommended stocking rate assumptions.

**Table S3.3** – Results of correlation analyses between Honeybee stocks and area of insect pollinated crops and pollination service capacity

| Variables | Coefficient (*ρ*) | P value |
| --- | --- | --- |
| HB 2005 x PSCL 2005 | -0.05 | 0.7584 |
| HB 2010 x PSCL 2010 | -0.01 | 0.9657 |
| HB 2005 x PSCA 2005 | -0.11 | 0.5109 |
| HB 2010 x PSCA 2010 | -0.06 | 0.6866 |
| HB 2005 x PSCU 2005 | -0.11 | 0.4755 |
| HB 2010 x PSCU 2010 | -0.08 | 0.6226 |
| Area 2005 x PSCL 2005 | -0.19 | 0.2348 |
| Area 2010 x PSCL 2010 | -0.24 | 0.1288 |
| Area 2005 x PSCA 2005 | -0.23 | 0.1409 |
| Area 2010 x PSCA 2010 | -0.28 | 0.0709 |
| Area 2005 x PSCU 2005 | -0.24 | 0.1264 |
| Area 2010 x PSCU 2010 | -0.29 | 0.0667 |

Variables = variables for which correlation was assessed, Coefficient (*ρ*) = the Spearman’s rank correlation coefficient, P value = the significance of the coefficient. In all analyses n=41. SD = Supply Density, the number of colonies available per hectare of insect-pollinated crop in 2005 or 2010. PSC = Pollination service capacity, the capacity of honeybee stocks to supply pollination services under lower (L), average (A) and upper (U) recommended stocking rate assumptions.

**Table S3.4** – Results of correlation analysis between supply factors and national insect pollinated crop characteristics among insect pollinated crop area

| Variables | Coefficient (r) | P value |
| --- | --- | --- |
| SD 2005 x OFR 2005 | -0.34 | 0.0293 |
| SD 2010 x OFR 2010 | -0.54 | <0.0001 |
| PSCL 2005 x OFR 2005 | -0.27 | 0.0884 |
| PSCL 2010 x OFR 2010 | -0.54 | <0.0001 |
| PSCA 2005 x OFR 2005 | -0.29 | 0.065 |
| PSCA 2010 x OFR 2010 | -0.51 | <0.0001 |
| PSCU 2005 x OFR 2005 | -0.29 | 0.0686 |
| PSCU 2010 x OFR 2010 | -0.50 | <0.0001 |
| SD 2005 x BF 2005 | -0.25 | 0.1167 |
| SD 2010 x BF 2010 | -0.30 | 0.0576 |
| PSCL 2005 x BF 2005 | -0.19 | 0.2254 |
| PSCL 2010 x BF 2010 | -0.29 | 0.0618 |
| PSCA 2005 x BF 2005 | -0.22 | 0.1615 |
| PSCA 2010 x BF 2010 | -0.28 | 0.0764 |
| PSCU 2005 x BF 2005 | -0.23 | 0.1473 |
| PSCU 2010 x BF 2010 | -0.28 | 0.0717 |

Variables = variables for which correlation was assessed, Coefficient (r) = the Pearson’s product moment correlation coefficient, P value = the significance of the coefficient. In all analyses n=41. SD = Supply Density, the number of colonies available per hectare of insect-pollinated crop in 2005 or 2010. PSC = Pollination service capacity, the capacity of honey bee stocks to supply pollination services under lower (L), average (A) and upper (U) recommended stocking rate assumptions. OFR = ratio of oilseeds to fruits within insect pollinated crop area. BF = total area of biofuel crops, oilseed rape, sunflower and soybean.

**Table S3.5 –** Results of correlation analysis between density of demand for pollination services and ratio of oilseeds to orchards among insect pollinated crop area

| Variables | Coefficient (*ρ*) | P value |
| --- | --- | --- |
| DDL 2005 x OFR 2005 | -0.36 | 0.0201 |
| DDL 2010 x OFR 2010 | -0.43 | 0.0046 |
| DDA 2005 x OFR 2005 | -0.63 | <0.0001 |
| DDA 2010 x OFR 2010 | -0.64 | <0.0001 |
| DDU 2005 x OFR 2005 | -0.68 | <0.0001 |
| DDU 2010 x OFR 2010 | -0.64 | <0.0001 |
| DDL 2005 x BF 2005 | -0.28 | 0.0793 |
| DDL 2010 x BF 2010 | -0.29 | 0.0645 |
| DDA 2005 x BF 2005 | -0.62 | <0.0001 |
| DDA 2010 x BF 2010 | -0.65 | <0.0001 |
| DDU 2005 x BF 2005 | -0.63 | <0.0001 |
| DDU 2010 x BF 2010 | -0.66 | <0.0001 |

Variables = variables for which correlation was assessed, Coefficient (r) = the Pearson’s product moment correlation coefficient, P value = the significance of the coefficient. In all analyses n=41. DD = Density of Demand, (the mean number of honey bee colonies required per hectare of insect pollinated crop, weighted by the area of each crop and divided by two to take into account the potential for hive movement within a year) at lower (L), average (A) and upper (U) recommended stocking rate assumptions. OFR = ratio of oilseeds to fruits within insect-pollinated crop area.

**Table S3.6 –** Results of correlation analysis between changes in density of demand and changes in other variables

| Variables | Coefficient (*ρ*) | P value |
| --- | --- | --- |
| Δ DDL x Δ SD | 0.4 | 0.0097 |
| Δ DDA x Δ SD | 0.07 | 0.6536 |
| Δ DDU x Δ SD | 0.08 | 0.6173 |
| Δ DDL x Δ PSCL | 0.27 | 0.0849 |
| Δ DDA x Δ PSCA | -0.01 | 0.9595 |
| Δ DDU x Δ PSCU | -0.06 | 0.7288 |
| Δ DDL x Δ OFR | -0.47 | 0.019 |
| Δ DDA x Δ OFR | -0.53 | <0.0001 |
| Δ DDU x Δ OFR | -0.67 | <0.0001 |
| Δ DDL x Δ BF | -0.53 | <0.0001 |
| Δ DDA x Δ BF | -0.44 | 0.0046 |
| Δ DDU x Δ BF | -0.44 | 0.0042 |

Variables = variables for which correlation was assessed, Coefficient (r) = the Pearson’s product moment correlation coefficient, P value = the significance of the coefficient. In all analyses n=41. Δ DD = % change in Density of Demand, (the mean number of honey bee colonies required per hectare of insect-pollinated crop, weighted by the area of each crop and divided by two to represent the potential for hive movement within a year) at lower (L), average (A) and upper (U) recommended stocking rate assumptions between 2005 and 2010. Δ SD = Supply Density, the number of colonies available per hectare of insect-pollinated crop between 2005 and 2010. Δ PSC = % change in Pollination service capacity, the capacity of honey bee stocks to supply pollination services under lower (L), average (A) and upper (U) recommended stocking rate assumptions. Δ OFR = % change in the ratio of oilseeds to fruits within insect-pollinated crop area between 2005 and 2010. Δ BF = % change in the total area of biofuel crops, oilseed rape, sunflower and soybean between 2005 and 2010. Note, as an outlier, Greece was excluded from all Δ BF analyses.

**Table S3.7 –** Results of correlation analysis between changes in supply factors and changes in other variables

| Variables | Coefficient (r) | P value |
| --- | --- | --- |
| Δ Area x Δ HB | -0.3 | 0.0548 |
| Δ Area x Δ OFR | 0.01 | 0.9584 |
| Δ SD x Δ OFR | -0.09 | 0.5946 |
| Δ PSCL x Δ OFR | -0.09 | 0.5782 |
| Δ PSCA x Δ OFR | -0.06 | 0.7146 |
| Δ PSCU x Δ OFR | -0.05 | 0.7703 |
| Δ Area x Δ BF | 0.79 | <0.0001 |
| Δ SD x Δ BF | -0.59 | <0.0001 |
| Δ PSCL x Δ BF | -0.52 | <0.0001 |
| Δ PSCA x Δ BF | -0.53 | <0.0001 |
| Δ PSCU x Δ BF | -0.52 | <0.0001 |
| Δ OOR x Δ BF | 0.45 | 0.0038 |

Variables = variables for which correlation was assessed, Coefficient (r) = the Pearson’s product moment correlation coefficient, P value = the significance of the coefficient. In all analyses n=41. Δ Area = % change in the total area of insect-pollinated crops between 2005 and 2010. Δ DD = % change in Density of Demand, (the mean number of honey bee colonies required per hectare of insect-pollinated crop, weighted by the area of each crop and divided by two to take into account the potential for hive movement within a year) at lower (L), average (A) and upper (U) recommended stocking rate assumptions between 2005 and 2010. Δ SD = Supply Density, the number of colonies available per hectare of insect-pollinated crop between 2005 and 2010. Δ PSC = % change in Pollination service capacity, the capacity of honeybee stocks to supply pollination services under lower (L), average (A) and upper (U) recommended stocking rate assumptions. Δ OFR = % change in the ratio of oilseeds to orchard fruits within insect-pollinated crop area between 2005 and 2010. Δ BF = % change in the total area of biofuel crops, oilseed rape, sunflower and soybean between 2005 and 2010. Note; Δ SD correlates perfectly with Δ PSC at all recommended stocking rates. Note, as an outlier, Greece was excluded from all Δ BF analyses.

**Table S3.8 –** Results of categorical regression analysis of the significance in variation between EU and non-EU member states

| Variables | Α | EU-12 | EU-15 |
| --- | --- | --- | --- |
| SD 2005 | 2.1536^***^ | -0.2061 | -0.5636 |
| SD 2010 | 2.7400^***^ | -1.0025 | -1.2400 |
| Log DDL 2005 | -0.5309^***^ | -0.0549 | 0.0355 |
| Log DDL 2010 | -0.5181^***^ | -0.0919 | -0.0046 |
| Log DDA 2005 | 0.4501^***^ | -0.0548 | 0.0581 |
| Log DDA 2010 | 0.4430^***^ | -0.0504 | 0.0338 |
| Log DDU 2005 | 1.1439^***^ | -0.0876 | -0.0106 |
| Log DDU 2010 | 1.1237^***^ | -0.1128 | -0.0320 |
| PSCL 2005 | 3.6136^*^ | 0.5673 | -0.9349 |
| PSCL 2010 | 4.4964^***^ | -2.6873^*^ | -1.9264^*^ |
| PSCA 2005 | 1.2436^***^ | -0.0269 | -0.3122 |
| PSCA 2010 | 1.6257^***^ | -0.5382 | -0.7337 |
| PSCU 2005 | 0.6214^***^ | -0.0256 | -0.1328 |
| PSCU 2005 | 0.8207^***^ | -0.2615 | -0.3487 |
| OFR 2005 | 2.312 | 5.029 | 4.815 |
| OFR 2010 | 3.077 | 8.372^*^ | 7.625^*^ |

Variables = Variable assessed, α = regression intercept coefficient, EU-15 = slope coefficient for the 15 pre-2004 members of the European Union, EU-12 = slope coefficient for members of the EU joining in or after 2004. Significance: ^***^ = P<0.0001, ^**^ = P<0.01, ^*^ = P<0.05, + = P<0.1. SD = Supply Density, the number of colonies available per hectare of insect-pollinated crop in 2005 or 2010. PSC = Pollination service capacity, the capacity of honey bee stocks to supply pollination services under lower (L), average (A) and upper (U) recommended stocking rate assumptions. OFR = ratio of oilseeds to fruits within insect pollinated crop area

**Table S3.9 –** Results of categorical regression analysis of the significance in variation between countries in different regions of Europe

| Variables | Α | North | South | West |
| --- | --- | --- | --- | --- |
| SD 2005 | 0.8367 | 0.5700 | 1.9827^*^ | 0.8876 |
| SD 2010 | 0.6744 | 0.5933 | 2.6756^***^ | 0.8441 |
| Log DDL 2005 | -0.5931^***^ | 0.1223 | 0.0864 | -0.0089 |
| Log DDL 2010 | -0.6054^***^ | 0.0749 | 0.1186^+^ | -0.0229 |
| Log DDA 2005 | 0.2741^***^ | 0.2347^**^ | 0.2586^***^ | 0.1684^*^ |
| Log DDA 2010 | 0.2821^***^ | 0.1862^**^ | 0.2357^***^ | 0.1501^*^ |
| Log DDU 2005 | 0.9417^***^ | 0.1962^+^ | 0.2468^**^ | 0.1955^+^ |
| Log DDU 2010 | 0.9134^***^ | 0.1495 | 0.2491^**^ | 0.2082^+^ |
| PSCL 2005 | 1.5189 | 0.7878 | 3.7624^+^ | 1.6254 |
| PSCL 2010 | 1.2467 | 0.9378 | 3.3127^**^ | 1.5219 |
| PSCA 2005 | 0.6389 | 0.1578 | 0.9630^+^ | 0.4225 |
| PSCA 2010 | 0.5067 | 0.2167 | 1.4727^**^ | 0.4148 |
| PSCU 2005 | 0.3356^+^ | 0.0678 | 0.4726^*^ | 0.1787 |
| PSCU 2010 | 0.2744^+^ | 0.0878 | 0.7512^***^ | 0.1741 |
| OFR 2005 | 10.507^***^ | -1.498 | -9.922^***^ | -4.457 |
| OFR 2010 | 14.244^***^ | 1.541 | -13.738^***^ | -5.297 |

Variables = Variable assessed, α = regression intercept coefficient, EU-15 = slope coefficient for the 15 pre-2004 members of the European Union, EU-12 = slope coefficient for members of the EU joining in or after 2004. Significance: *** = P<0.0001, ** = P<0.01, * = P<0.05 + = P<0.1. SD = Supply Density, the number of colonies available per hectare of insect-pollinated crop in 2005 or 2010. PSC = Pollination service capacity, the capacity of honeybee stocks to supply pollination services under lower (L), average (A) and upper (U) recommended stocking rate assumptions. OFR = ratio of oilseeds to fruits within insect pollinated crop area

**Table S3.10 –** Results of categorical regression analysis of the significance in variation between EU and non-EU member states

| Variables | α | EU-12 | EU-15 |
| --- | --- | --- | --- |
| Δ Area | 0.0520 | 0.2090^+^ | 0.1489 |
| Δ HB | 0.2436^*^ | -0.1526 | -0.1535 |
| Δ SD | 0.4236 | -0.4494^+^ | -0.4709 |
| Δ PSCL | 0.3821^+^ | -0.3921 | -0.4081 |
| Δ PSCA | 0.4136 | -0.4394 | -0.4316 |
| Δ PSCU | 0.4607^*^ | -0.4499 | -0.4640 |
| Δ OOR | 0.1062 | 0.8323 | 1.5749 |
| Δ BF | 0.0535 | 0.4874* | 0.3779^+^ |

Variables = Variable assessed, α = regression intercept coefficient, EU-15 = slope coefficient for the 15 pre-2004 members of the European Union, EU-12 = slope coefficient for members of the EU joining in or after 2004. Significance: *** = P<0.0001, ** = P<0.01, * = P<0.05 + = P<0.1. Δ Area = % change in the total area of insect-pollinated crops between 2005 and 2010. Δ Area = % change in national honeybee stocks. Δ DD = % change in Density of Demand, (the mean number of hives required per hectare of insect pollinated crop, weighted by the area of each crop and divided by two to represent the potential for hive movement within a year) at lower (L), average (A) and upper (U) recommended stocking rate assumptions between 2005 and 2010. Δ SD = Supply Density, the number of colonies available per hectare of insect-pollinated crop between 2005 and 2010. Δ PSC = % change in Pollination service capacity, the capacity of honeybee stocks to supply pollination services under lower (L), average (A) and upper (U) recommended stocking rate assumptions. Δ OFR = % change in the ratio of oilseeds to orchard fruits within insect-pollinated crop area between 2005 and 2010. Δ BF = % change in the total area of biofuel crops, oilseed rape, sunflower and soybean between 2005 and 2010.

**Table S3.11 –** Results of categorical regression analysis of the significance in variation between different regions of Europe

| Variables | Α | North | South | West |
| --- | --- | --- | --- | --- |
| Δ Area | 0.2832^**^ | 0.1833 | -0.3318^**^ | -0.1544 |
| Δ HB | 0.0548 | 0.0294 | 0.2552^+^ | -0.1056 |
| Δ SD | -0.1600 | -0.0611 | 0.7393^*^ | 0.0271 |
| Δ PSCL | -0.1456 | -0.0289 | 0.6768^*^ | 0.0341 |
| Δ PSCA | -0.1656 | -0.0211 | 0.7481^*^ | 0.0312 |
| Δ PSCU | -0.1333 | -0.0300 | 0.7602^*^ | 0.0148 |
| Δ OFR | 0.5887 | 0.5446 | 0.3684 | 0.4336 |
| Δ BF | -0.1485 | 0.2667 | -0.557 | 0.5811 |

Variables = Variable assessed, α = regression intercept coefficient, EU-15 = slope coefficient for the 15 pre-2004 members of the European Union, EU-12 = slope coefficient for members of the EU joining in or after 2004. Significance: *** = P<0.0001, ** = P<0.01, * = P<0.05 + = P<0.1. Δ Area = % change in the total area of insect-pollinated crops between 2005 and 2010. Δ DD = % change in Density of Demand, (the mean number of hives required per hectare of insect-pollinated crop, weighted by the area of each crop and divided by two to represent the potential for hive movement within a year) at lower (L), average (A) and upper (U) recommended stocking rate assumptions between 2005 and 2010. Δ SD = Supply Density, the number of colonies available per hectare of insect-pollinated crop between 2005 and 2010. Δ PSC = % change in Pollination service capacity, the capacity of honey bee stocks to supply pollination services under lower (L), average (A) and upper (U) recommended stocking rate assumptions. Δ OFR = % change in the ratio of oilseeds to fruits within insect pollinated crop area between 2005 and 2010. Δ BF = % change in the total area of biofuel crops, oilseed rape, sunflower and soybean between 2005 and 2010.
