# Supplementary material for: Agricultural Policies Exacerbate Honeybee Pollination Service Supply-Demand Mismatches Across Europe
Source: PLoS One. 2014 Jan 8;9(1):e82996. doi: 10.1371/journal.pone.0082996 (PMC3885438; doi:10.1371/journal.pone.0082996)

**Supplemental S4 – Analysis under lower and upper Recommended Stocking Rates**

Lower and upper recommended stocking rates (RSR) respectively represent the lowest and highest numbers of colonies recommended by the cited sources in Supplemental S2 for providing adequate pollination services per hectare of crops. These estimates form the lower and upper bounds of density of demand (DD) and pollination service capacity (PSC) this study. In common with estimates under average RSR, DD within each year correlates negatively with the ratio of oilseeds to fruit crops under both lower (*ρ*= -0.36, p=0.020 and *ρ*= -0.43, p=0.005) and upper RSR (*ρ*= -0.68 and *ρ*= -0.64, p=<0.001). Under lower RRS DD is highest in Portugal (0.85 colonies/ha and 0.84 colonies/ha in 2005 and 2010 respectively), largely due to the influence of tree fruit and nut crops, such as almond which has high (2.5 colonies/ha) RSR compared to other crops. Under upper RSR, like average RSR, the Netherlands has the highest DD in both years (5.28 colonies/ha and 5.31 colonies/ha) due to its low areas of oilseeds and relative to more demanding fruit crops.

Under lower RSR assumptions, the pollination service capacity of honeybee stocks is inadequate to supply >90% of demands in only 5 countries in 2005, however, this rises to 11 countries by 2011 (Figure S1), notably in Ukraine, Latvia and Estonia where PSC declined substantially with mass expansion of oilseeds. Using upper RSR assumptions, however, 33 countries have deficits (<90%) in honeybee pollination service capacity in 2005, falling slightly to 32 by 2010 (Figure S2). The number of countries where honeybee stocks cannot provide >25% PSC however rises from 12 in 2005 to 14 in 2010. In total, European demands for honeybee pollination services rise by ~4M under lower RSR, 2.23 times the increase in honeybee colonies, and ~14.1M under upper RSR, 7.72 times the rate of honeybee colony growth. Consequently, total PSC for Europe as a whole falls from 172% to 161% under lower RSR and from 35% to 34% under upper RSR.

These findings highlight the high degree of variance within the RSR values available within the literature. In general however fruit crops tend to have higher demands per hectare than oilseeds, although there is less difference towards the lower RSR. The lowest RSR used within this study are often below 1 colony per hectare, resulting in demand being more closely tied to the area of crops than the specific demands for the crops themselves. At higher demand estimates, however, the impact of particular crops becomes much more significant, notably apple which is cultivated across every country surveyed and has an upper RSR of 12.5 colonies/ha.

Under all RSR, the findings demonstrate near-identical patterns of declining pollination service capacity from honeybee stocks, driven by a combination of honeybee declines and rising demands from expanded oilseed crop cultivations. Although the lower and upper RSR assumptions may be biased by potentially unrealistic estimates they nonetheless demonstrate that several countries have particular deficits (UK, Moldova, Finland, Lithuania, Serbia) or surpluses (Slovenia, Bosnia & Herzegovina, Portugal and Norway) under all assumptions.

**Figure S1.** A comparison of the density of demand (a,b) and the resultant pollination service capacity (c,d) in 2005 (left panels) and 2010 (right panels). Figures based on lower recommended stocking rate


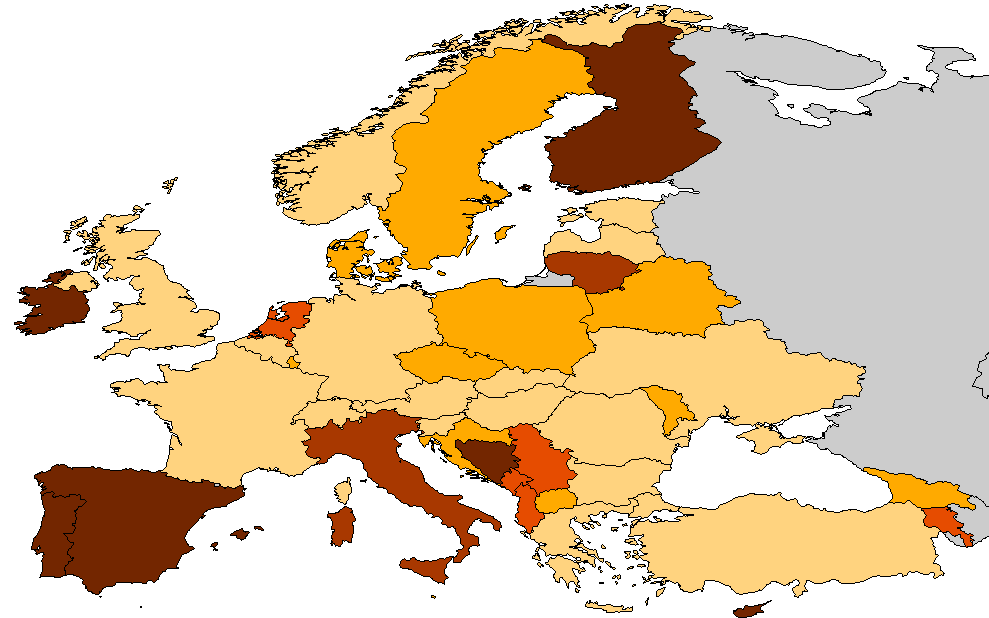

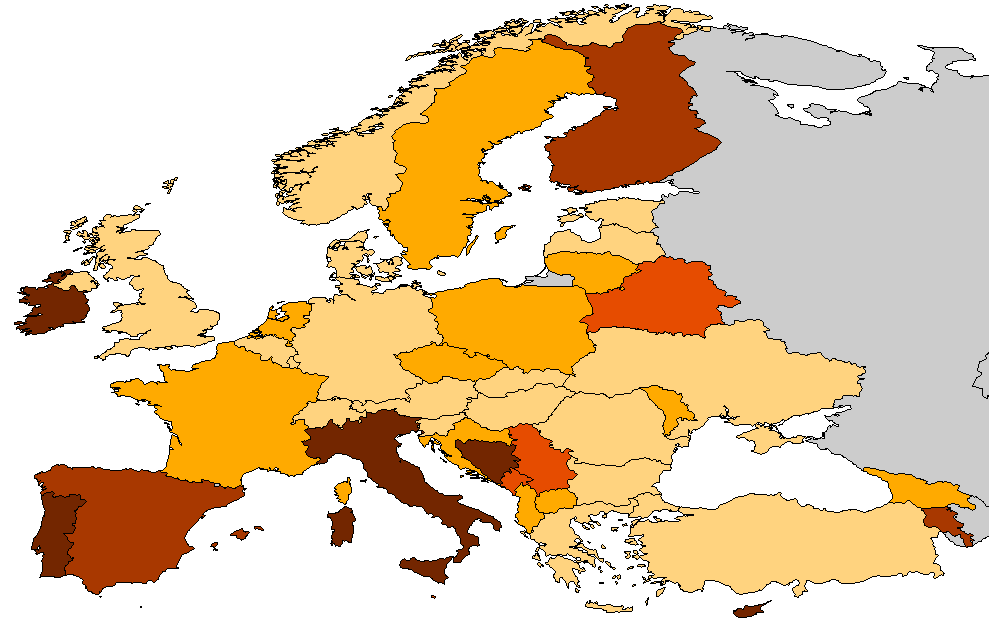


b

a


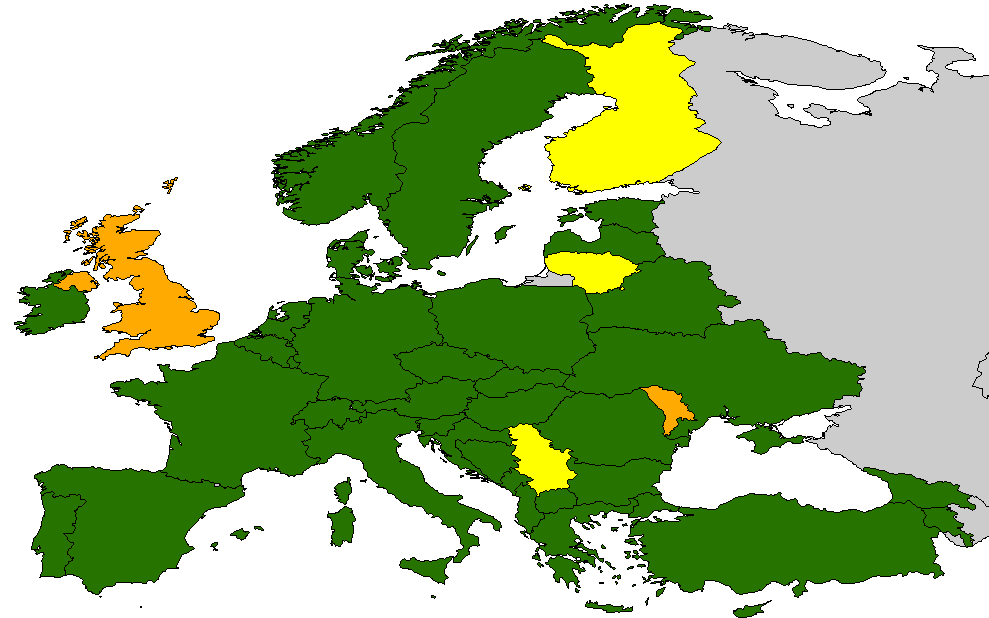

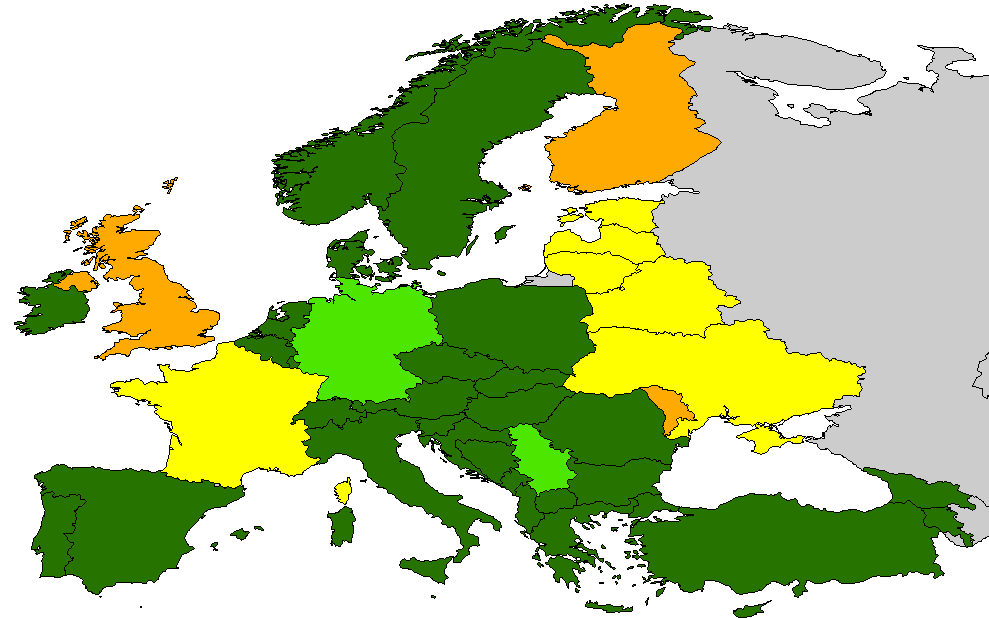


PSC: % Supply of honeybees relative to demand
>90%
75 – 90%
50 – 75%
25 – 50%
<25%
NA

DD: Mean colonies required/ha
>0.70
0.65 – 0.70
0.60 – 0.65
0.55 – 0.60
<0.55
NA

d

c


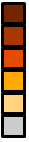

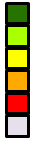


**Figure S2.** A comparison of the density of demand (a,b) and the resultant pollination service capacity (c,d) in 2005 (left panels) and 2010 (right panels). Figures based on upper recommended stocking rate


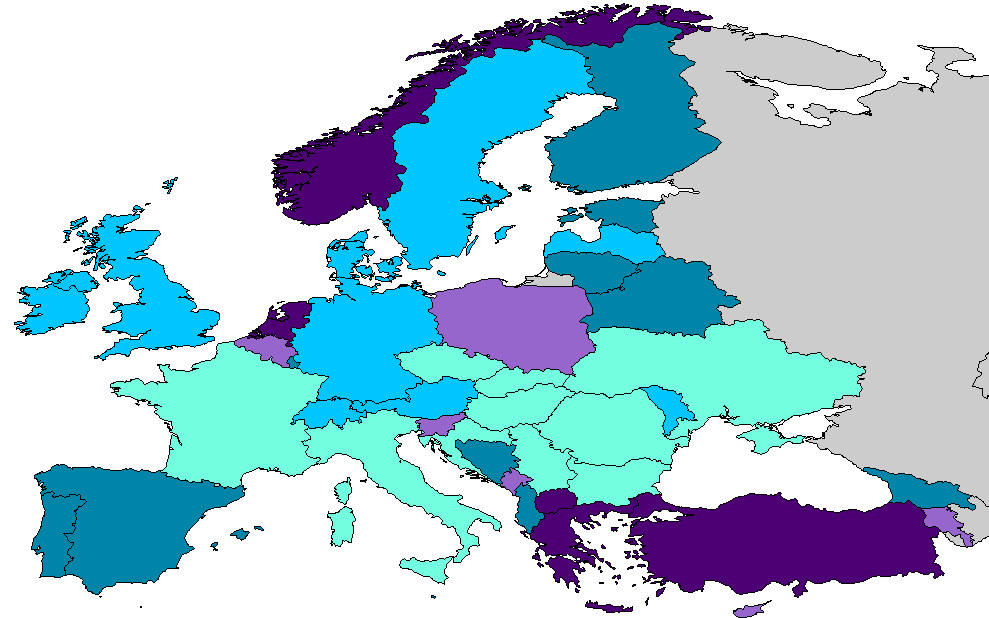

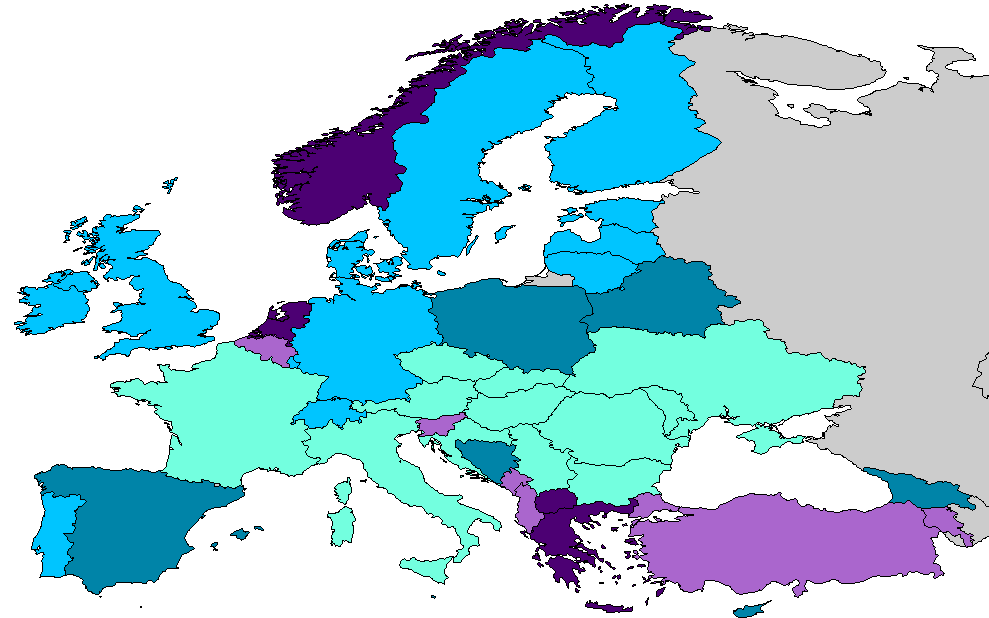


b

a


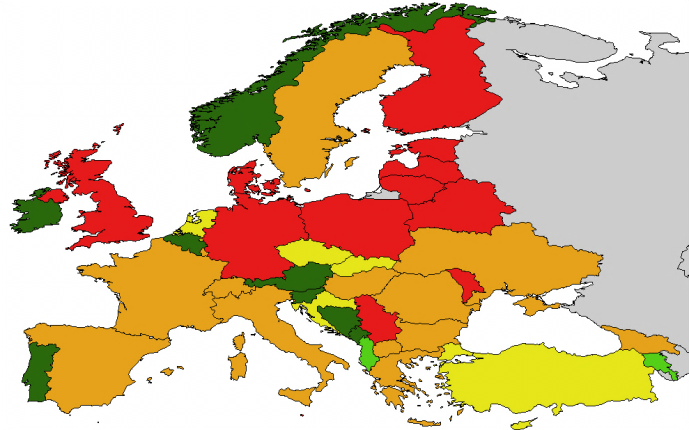

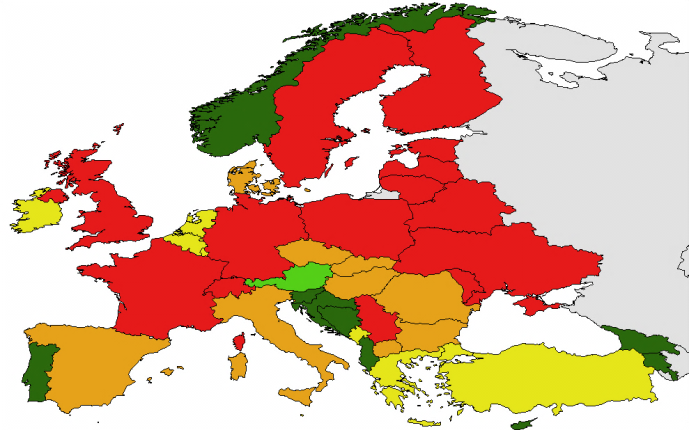


PSC: % Supply of honeybees relative to demand
>90%
75 – 90%
50 – 75%
25 – 50%
<25%
NA

DD: Mean colonies required/ha
>4.0
3.5 – 4.0
3.0 – 3.5
2.5 – 3.0
<2.5
NA

d

c


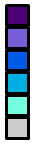

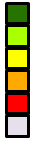

Supplement: Supporting Information S4 — Analysis under lower and upper Recommended Stocking Rates. (DOCX) [file pone.0082996.s004.docx]
