# Supplementary material for: Agricultural Policies Exacerbate Honeybee Pollination Service Supply-Demand Mismatches Across Europe
Source: PLoS One. 2014 Jan 8;9(1):e82996. doi: 10.1371/journal.pone.0082996 (PMC3885438; doi:10.1371/journal.pone.0082996)
Supplement: Supporting Information S5 — Summary of National Scale Results. (DOCX) [file pone.0082996.s005.docx]

**Supplemental S5 – Summary of National Scale Results**

**Table S5.1** – National Honeybee stock and insect-pollinated crop area data for 2005 and 2010

|  | Honeybee colonies (000) | | Area (000 Ha) | | % Oilseeds | | % orchard fruit | |
| --- | --- | --- | --- | --- | --- | --- | --- | --- |
| Country | 2005 | 2010 | 2005 | 2010 | 2005 | 2010 | 2005 | 2010 |
| Albania | 157.0 | 218.0 | 55.2 | 64.6 | 2% | 2% | 32% | 44% |
| Armenia | 113.1 | 203.6 | 37.3 | 38.6 | 0% | 0% | 76% | 73% |
| Austria | 291.4 | 367.6 | 121.8 | 161.6 | 54% | 49% | 6% | 5% |
| Belarus | 179.5 | 224.8 | 326.0 | 546.8 | 42% | 62% | 27% | 15% |
| Belgium | 100.0 | 112.0 | 30.4 | 45.4 | 19% | 25% | 53% | 57% |
| Bosnia & Herzegovina | 259.6 | 366.6 | 62.0 | 63.1 | 1% | 2% | 59% | 66% |
| Bulgaria | 660.0 | 613.3 | 711.9 | 1,021.3 | 94% | 93% | 3% | 3% |
| Croatia | 167.0 | 308.0 | 143.8 | 127.1 | 49% | 34% | 11% | 19% |
| Cyprus | 40.5 | 44.0 | 17.6 | 10.7 | 0% | 0% | 88% | 93% |
| Czech Republic | 551.7 | 528.2 | 422.7 | 506.1 | 73% | 78% | 5% | 3% |
| Denmark | 82.0 | 170.0 | 126.8 | 179.6 | 88% | 93% | 3% | 2% |
| Estonia | 33.0 | 28.0 | 58.8 | 103.8 | 79% | 93% | 15% | 5% |
| Finland | 50.0 | 46.0 | 97.7 | 186.9 | 78% | 84% | 1% | 0% |
| France | 1,398.4 | 1,074.2 | 2,249.9 | 2,559.6 | 83% | 84% | 6% | 5% |
| Georgia | 149.5 | 311.5 | 136.9 | 52.2 | 27% | 17% | 33% | 28% |
| Germany | 750.0 | 711.9 | 1,477.9 | 1,589.4 | 93% | 94% | 3% | 3% |
| Greece | 1,313.7 | 1,546.6 | 604.9 | 543.6 | 1% | 14% | 28% | 30% |
| Hungary | 910.9 | 997.0 | 815.4 | 922.7 | 79% | 82% | 11% | 9% |
| Italy | 1,000.0 | 1,127.8 | 982.7 | 943.0 | 14% | 13% | 53% | 51% |
| Ireland | 22.0 | 24.0 | 7.2 | 12.0 | 51% | 67% | 9% | 5% |
| Latvia | 52.4 | 37.8 | 85.4 | 119.4 | 84% | 93% | 13% | 4% |
| Lithuania | 82.8 | 102.6 | 180.4 | 306.9 | 61% | 84% | 13% | 5% |
| Luxembourg | 8.1 | 8.2 | 6.8 | 5.1 | 68% | 93% | 30% | 6% |
| Malta | 1.3 | 2.7 | 1.8 | 1.5 | 0% | 0% | 26% | 28% |
| Macedonia | 66.7 | 76.1 | 41.8 | 41.5 | 13% | 10% | 25% | 32% |
| Moldova | 87.0 | 102.0 | 446.5 | 461.4 | 62% | 63% | 22% | 19% |
| Montenegro | 42.6 | 21.7 | 7.9 | 9.2 | 0% | 0% | 62% | 66% |
| Netherlands | 80.0 | 80.0 | 26.1 | 27.5 | 9% | 11% | 67% | 65% |
| Norway | 64.5 | 64.5 | 11.5 | 10.1 | 58% | 59% | 22% | 22% |
| Poland | 949.2 | 1,123.4 | 1,047.7 | 1,443.4 | 53% | 66% | 24% | 17% |
| Portugal | 632.5 | 562.6 | 205.8 | 184.3 | 3% | 8% | 75% | 73% |
| Romania | 930.0 | 1,101.0 | 1,485.3 | 1,610.2 | 71% | 82% | 14% | 9% |
| Serbia | 270.0 | 320.0 | 563.0 | 575.8 | 35% | 32% | 28% | 27% |
| Slovakia | 276.5 | 235.7 | 221.1 | 275.3 | 89% | 90% | 3% | 3% |
| Slovenia | 164.8 | 158.1 | 13.2 | 17.7 | 17% | 31% | 33% | 21% |
| Spain | 2,238.1 | 2,459.4 | 2,056.6 | 2,011.5 | 25% | 35% | 55% | 50% |
| Sweden | 84.3 | 77.4 | 96.4 | 132.2 | 85% | 83% | 2% | 2% |
| Switzerland | 31.7 | 16.9 | 31.7 | 33.8 | 72% | 75% | 21% | 19% |
| Turkey | 4,590.0 | 5,602.7 | 1,920.3 | 1,980.4 | 30% | 34% | 20% | 17% |
| United Kingdom | 289.8 | 274.0 | 809.8 | 882.7 | 73% | 77% | 2% | 2% |
| Ukraine | 3,369.0 | 2,921.5 | 5,305.2 | 7,255.5 | 73% | 74% | 4% | 3% |
| Europe | 22,540.3 | 24,371.2 | 23,050.3 | 27,063.2 | 60% | 17% | 66% | 13% |

Area = Sum area of all insect pollinated crops for which honeybees are known to be effective pollinators, % Oilseeds – the proportion of the total crop area occupied by oilseed crops, % Orchard Fruit = the proportion of crop area occupied by orchard fruits

**Table S5.2** – National supply and demand data under lower Recommended Stocking Rate assumptions

|  | Supply Density (colonies/ha) | | Total Demand (000 colonies) | | Density of Demand (colonies/ha/2) | | Pollination Service Capacity | |
| --- | --- | --- | --- | --- | --- | --- | --- | --- |
| Country | 2005 | 2010 | 2005 | 2010 | 2005 | 2010 | 2005 | 2010 |
| Albania | 2.84 | 3.38 | 70.3 | 78.1 | 0.64 | 0.60 | 446% | 558% |
| Armenia | 3.03 | 5.27 | 46.8 | 52.5 | 0.63 | 0.68 | 483% | 775% |
| Austria | 2.39 | 2.27 | 125.7 | 164.0 | 0.52 | 0.51 | 464% | 448% |
| Belarus | 0.55 | 0.41 | 381.9 | 672.0 | 0.59 | 0.61 | 94% | 67% |
| Belgium | 3.29 | 2.47 | 31.6 | 46.6 | 0.52 | 0.51 | 634% | 429% |
| Bosnia & Herzegovina | 4.19 | 5.81 | 97.8 | 100.5 | 0.79 | 0.80 | 531% | 730% |
| Bulgaria | 0.93 | 0.60 | 727.1 | 1,013.1 | 0.51 | 0.50 | 182% | 121% |
| Croatia | 1.16 | 2.42 | 173.8 | 153.2 | 0.60 | 0.60 | 192% | 402% |
| Cyprus | 2.30 | 4.13 | 26.2 | 16.5 | 0.74 | 0.77 | 309% | 534% |
| Czech Republic | 1.31 | 1.04 | 494.8 | 574.0 | 0.59 | 0.57 | 223% | 184% |
| Denmark | 0.65 | 0.95 | 142.0 | 191.4 | 0.56 | 0.53 | 116% | 178% |
| Estonia | 0.56 | 0.27 | 65.2 | 107.9 | 0.55 | 0.52 | 101% | 52% |
| Finland | 0.51 | 0.25 | 157.3 | 257.3 | 0.80 | 0.69 | 67% | 36% |
| France | 0.62 | 0.42 | 2,496.2 | 2,873.6 | 0.55 | 0.56 | 112% | 75% |
| Georgia | 1.09 | 5.97 | 152.2 | 62.6 | 0.56 | 0.60 | 196% | 994% |
| Germany | 0.51 | 0.45 | 1,536.7 | 1,633.6 | 0.52 | 0.51 | 98% | 87% |
| Greece | 2.17 | 2.85 | 459.2 | 464.9 | 0.38 | 0.43 | 572% | 665% |
| Hungary | 1.12 | 1.08 | 847.4 | 961.4 | 0.52 | 0.52 | 215% | 207% |
| Italy | 1.02 | 1.20 | 1,368.7 | 1,338.6 | 0.70 | 0.71 | 146% | 169% |
| Ireland | 3.04 | 2.00 | 11.5 | 17.0 | 0.79 | 0.71 | 382% | 282% |
| Latvia | 0.61 | 0.32 | 89.3 | 123.3 | 0.52 | 0.52 | 117% | 61% |
| Lithuania | 0.46 | 0.33 | 240.3 | 351.8 | 0.67 | 0.57 | 69% | 58% |
| Luxembourg | 1.19 | 1.61 | 7.8 | 5.2 | 0.57 | 0.51 | 207% | 316% |
| Malta | 0.76 | 1.83 | 2.1 | 1.9 | 0.59 | 0.63 | 130% | 292% |
| Macedonia | 1.62 | 1.83 | 47.3 | 49.2 | 0.58 | 0.59 | 282% | 309% |
| Moldova | 0.19 | 0.22 | 503.3 | 518.1 | 0.56 | 0.56 | 35% | 39% |
| Montenegro | 5.37 | 2.37 | 10.0 | 11.7 | 0.63 | 0.64 | 851% | 372% |
| Netherlands | 3.07 | 2.91 | 31.8 | 32.6 | 0.61 | 0.59 | 503% | 491% |
| Norway | 5.60 | 6.39 | 12.6 | 11.2 | 0.55 | 0.55 | 1022% | 1152% |
| Poland | 0.91 | 0.78 | 1,220.8 | 1,625.0 | 0.58 | 0.56 | 156% | 138% |
| Portugal | 3.07 | 3.05 | 350.7 | 309.3 | 0.85 | 0.84 | 361% | 364% |
| Romania | 0.63 | 0.68 | 1,648.2 | 1,703.5 | 0.55 | 0.53 | 113% | 129% |
| Serbia | 0.48 | 0.56 | 715.3 | 748.2 | 0.64 | 0.65 | 75% | 86% |
| Slovakia | 1.25 | 0.86 | 234.9 | 289.6 | 0.53 | 0.53 | 235% | 163% |
| Slovenia | 12.53 | 8.93 | 10.4 | 13.6 | 0.40 | 0.38 | 3167% | 2332% |
| Spain | 1.09 | 1.22 | 2,910.7 | 2,759.7 | 0.71 | 0.69 | 154% | 178% |
| Sweden | 0.87 | 0.59 | 110.0 | 157.1 | 0.57 | 0.59 | 153% | 99% |
| Switzerland | 1.00 | 0.50 | 34.7 | 37.0 | 0.55 | 0.55 | 183% | 92% |
| Turkey | 2.39 | 2.83 | 1,654.3 | 1,738.1 | 0.43 | 0.44 | 555% | 645% |
| UK | 0.36 | 0.31 | 1,095.8 | 1,144.1 | 0.68 | 0.65 | 49% | 48% |
| Ukraine | 0.64 | 0.40 | 5,885.2 | 7,901.9 | 0.55 | 0.54 | 114% | 74% |
| Europe | 0.98 | 0.90 | 26,228.1 | 30,310.7 | 0.57 | 0.56 | 172% | 161% |

Supply Density = available honeybee colonies per hectare of insect pollinated crop, Total Demand = total number of colonies demanded under lower RSR assumptions. Density of demand = mean number of colonies required per hectare divided by two to take into account potential colony movement within the year. Pollination service capacity = maximum capacity of honeybee stocks to supply pollination services, taken as supply density divided by density of demand under lower recommended stocking rate assumptions

**Table S5.3** – National supply and demand data under average Recommended Stocking Rate assumptions

|  | Supply Density (colonies/ha) | | Total Demand (000 colonies) | | Density of Demand (colonies/ha) | | Pollination Service Capacity | |
| --- | --- | --- | --- | --- | --- | --- | --- | --- |
| Country | 2005 | 2010 | 2005 | 2010 | 2005 | 2010 | 2005 | 2010 |
| Albania | 2.84 | 3.38 | 195.8 | 221.9 | 1.77 | 1.72 | 160% | 196% |
| Armenia | 3.03 | 5.27 | 137.2 | 147.5 | 1.84 | 1.91 | 165% | 276% |
| Austria | 2.39 | 2.27 | 330.3 | 440.9 | 1.36 | 1.36 | 176% | 167% |
| Belarus | 0.55 | 0.41 | 999.2 | 1708.7 | 1.53 | 1.56 | 36% | 26% |
| Belgium | 3.29 | 2.47 | 102.7 | 149.2 | 1.69 | 1.64 | 195% | 134% |
| Bosnia & Herzegovina | 4.19 | 5.81 | 224.1 | 220.8 | 1.81 | 1.75 | 232% | 332% |
| Bulgaria | 0.93 | 0.60 | 1596.4 | 2452.0 | 1.12 | 1.20 | 83% | 50% |
| Croatia | 1.16 | 2.42 | 328.0 | 278.9 | 1.14 | 1.10 | 102% | 221% |
| Cyprus | 2.30 | 4.13 | 70.5 | 42.2 | 2.00 | 1.98 | 115% | 208% |
| Czech Republic | 1.31 | 1.04 | 1199.0 | 1441.2 | 1.42 | 1.42 | 92% | 73% |
| Denmark | 0.65 | 0.95 | 398.4 | 548.6 | 1.57 | 1.53 | 41% | 62% |
| Estonia | 0.56 | 0.27 | 184.2 | 310.8 | 1.56 | 1.50 | 36% | 18% |
| Finland | 0.51 | 0.25 | 370.2 | 633.0 | 1.89 | 1.69 | 27% | 15% |
| France | 0.62 | 0.42 | 6102.7 | 7015.9 | 1.36 | 1.37 | 46% | 31% |
| Georgia | 1.09 | 5.97 | 409.2 | 166.6 | 1.50 | 1.60 | 73% | 374% |
| Germany | 0.51 | 0.45 | 4417.4 | 4744.0 | 1.49 | 1.49 | 34% | 30% |
| Greece | 2.17 | 2.85 | 2540.7 | 2108.5 | 2.10 | 1.94 | 103% | 147% |
| Hungary | 1.12 | 1.08 | 1990.5 | 2288.3 | 1.22 | 1.24 | 92% | 87% |
| Italy | 1.02 | 1.20 | 2902.6 | 2797.9 | 1.48 | 1.48 | 69% | 81% |
| Ireland | 3.04 | 2.00 | 24.4 | 38.5 | 1.68 | 1.60 | 181% | 125% |
| Latvia | 0.61 | 0.32 | 262.8 | 356.2 | 1.54 | 1.49 | 40% | 21% |
| Lithuania | 0.46 | 0.33 | 595.3 | 937.7 | 1.65 | 1.53 | 28% | 22% |
| Luxembourg | 1.19 | 1.61 | 21.2 | 14.9 | 1.55 | 1.47 | 76% | 109% |
| Malta | 0.76 | 1.83 | 5.6 | 4.9 | 1.59 | 1.64 | 48% | 112% |
| Macedonia | 1.62 | 1.83 | 165.2 | 167.9 | 2.01 | 2.02 | 81% | 91% |
| Moldova | 0.19 | 0.22 | 1108.9 | 1123.4 | 1.24 | 1.22 | 16% | 18% |
| Montenegro | 5.37 | 2.37 | 28.8 | 32.7 | 1.81 | 1.79 | 296% | 133% |
| Netherlands | 3.07 | 2.91 | 109.5 | 114.1 | 2.10 | 2.08 | 146% | 140% |
| Norway | 5.60 | 6.39 | 44.8 | 38.9 | 1.95 | 1.93 | 287% | 331% |
| Poland | 0.91 | 0.78 | 3731.2 | 4757.4 | 1.78 | 1.65 | 51% | 47% |
| Portugal | 3.07 | 3.05 | 755.2 | 638.7 | 1.83 | 1.73 | 168% | 176% |
| Romania | 0.63 | 0.68 | 3571.7 | 4155.7 | 1.20 | 1.29 | 52% | 53% |
| Serbia | 0.48 | 0.56 | 1457.8 | 1460.9 | 1.29 | 1.27 | 37% | 44% |
| Slovakia | 1.25 | 0.86 | 560.0 | 719.0 | 1.27 | 1.31 | 99% | 66% |
| Slovenia | 12.53 | 8.93 | 45.5 | 60.2 | 1.73 | 1.70 | 724% | 525% |
| Spain | 1.09 | 1.22 | 7753.6 | 7188.0 | 1.89 | 1.79 | 58% | 68% |
| Sweden | 0.87 | 0.59 | 309.1 | 420.6 | 1.60 | 1.59 | 55% | 37% |
| Switzerland | 1.00 | 0.50 | 92.2 | 100.3 | 1.46 | 1.48 | 69% | 34% |
| Turkey | 2.39 | 2.83 | 6971.5 | 6843.6 | 1.82 | 1.73 | 132% | 164% |
| UK | 0.36 | 0.31 | 2554.8 | 2754.0 | 1.58 | 1.56 | 21% | 20% |
| Ukraine | 0.64 | 0.40 | 12475.0 | 16455.5 | 1.18 | 1.13 | 54% | 36% |
| Europe | 0.98 | 0.90 | 67,143.1 | 76,099.9 | 1.46 | 1.41 | 66% | 64% |

Supply Density = available honeybee colonies per hectare of insect pollinated crop, Total Demand = total number of colonies demanded under average RSR assumptions. Density of demand = mean number of colonies required per hectare divided by two to represent colony movement within the year. Pollination service capacity = maximum capacity of honeybee stocks to supply pollination services, taken as supply density divided by density of demand under average recommended stocking rate assumptions

**Table S5.4** – National supply and demand data under upper Recommended Stocking Rate assumptions

|  | Supply Density (colonies/ha) | | Total Demand (000 colonies) | | Density of Demand (colonies/ha) | | Pollination Service Capacity | |
| --- | --- | --- | --- | --- | --- | --- | --- | --- |
| Country | 2005 | 2010 | 2005 | 2010 | 2005 | 2010 | 2005 | 2010 |
| Albania | 2.84 | 3.38 | 371.4 | 456.1 | 3.36 | 3.53 | 85% | 96% |
| Armenia | 3.03 | 5.27 | 287.1 | 318.3 | 3.85 | 4.12 | 79% | 128% |
| Austria | 2.39 | 2.27 | 627.8 | 817.8 | 2.58 | 2.53 | 93% | 90% |
| Belarus | 0.55 | 0.41 | 2,147.0 | 3,264.7 | 3.29 | 2.99 | 17% | 14% |
| Belgium | 3.29 | 2.47 | 220.6 | 354.5 | 3.63 | 3.90 | 91% | 56% |
| Bosnia & Herzegovina | 4.19 | 5.81 | 392.9 | 386.1 | 3.17 | 3.06 | 132% | 190% |
| Bulgaria | 0.93 | 0.60 | 3,036.2 | 4,606.3 | 2.13 | 2.26 | 43% | 27% |
| Croatia | 1.16 | 2.42 | 561.8 | 474.4 | 1.95 | 1.87 | 59% | 130% |
| Cyprus | 2.30 | 4.13 | 124.5 | 71.6 | 3.54 | 3.36 | 65% | 123% |
| Czech Republic | 1.31 | 1.04 | 2,077.9 | 2,473.9 | 2.46 | 2.44 | 53% | 43% |
| Denmark | 0.65 | 0.95 | 732.3 | 987.5 | 2.89 | 2.75 | 22% | 34% |
| Estonia | 0.56 | 0.27 | 362.4 | 558.5 | 3.08 | 2.69 | 18% | 10% |
| Finland | 0.51 | 0.25 | 639.6 | 1,078.9 | 3.27 | 2.89 | 16% | 9% |
| France | 0.62 | 0.42 | 10,843.2 | 12,367.1 | 2.41 | 2.42 | 26% | 17% |
| Georgia | 1.09 | 5.97 | 889.1 | 321.4 | 3.25 | 3.08 | 34% | 194% |
| Germany | 0.51 | 0.45 | 7,936.7 | 8,512.9 | 2.69 | 2.68 | 19% | 17% |
| Greece | 2.17 | 2.85 | 5,827.0 | 4,663.7 | 4.82 | 4.29 | 45% | 66% |
| Hungary | 1.12 | 1.08 | 3,874.0 | 4,328.9 | 2.38 | 2.35 | 47% | 46% |
| Italy | 1.02 | 1.20 | 4,864.8 | 4,644.1 | 2.48 | 2.46 | 41% | 49% |
| Ireland | 3.04 | 2.00 | 42.4 | 65.7 | 2.93 | 2.74 | 104% | 73% |
| Latvia | 0.61 | 0.32 | 511.1 | 646.0 | 2.99 | 2.70 | 21% | 12% |
| Lithuania | 0.46 | 0.33 | 1,134.0 | 1,667.7 | 3.14 | 2.72 | 15% | 12% |
| Luxembourg | 1.19 | 1.61 | 41.7 | 27.2 | 3.07 | 2.68 | 39% | 60% |
| Malta | 0.76 | 1.83 | 11.2 | 9.5 | 3.18 | 3.19 | 24% | 57% |
| Macedonia | 1.62 | 1.83 | 342.0 | 353.2 | 4.15 | 4.25 | 39% | 43% |
| Moldova | 0.19 | 0.22 | 2,350.9 | 2,302.6 | 2.63 | 2.50 | 7% | 9% |
| Montenegro | 5.37 | 2.37 | 56.6 | 65.3 | 3.57 | 3.57 | 151% | 66% |
| Netherlands | 3.07 | 2.91 | 275.2 | 281.7 | 5.28 | 5.13 | 58% | 57% |
| Norway | 5.60 | 6.39 | 103.9 | 89.3 | 4.51 | 4.43 | 124% | 144% |
| Poland | 0.91 | 0.78 | 7,998.0 | 9,583.1 | 3.82 | 3.32 | 24% | 23% |
| Portugal | 3.07 | 3.05 | 1,237.2 | 1,030.5 | 3.01 | 2.80 | 102% | 109% |
| Romania | 0.63 | 0.68 | 6,964.4 | 7,844.1 | 2.34 | 2.44 | 27% | 28% |
| Serbia | 0.48 | 0.56 | 2,632.4 | 2,622.0 | 2.34 | 2.28 | 21% | 24% |
| Slovakia | 1.25 | 0.86 | 1,007.4 | 1,265.0 | 2.28 | 2.30 | 55% | 37% |
| Slovenia | 12.53 | 8.93 | 102.1 | 128.6 | 3.88 | 3.63 | 323% | 246% |
| Spain | 1.09 | 1.22 | 13,046.5 | 12,200.7 | 3.17 | 3.03 | 34% | 40% |
| Sweden | 0.87 | 0.59 | 568.5 | 756.3 | 2.95 | 2.86 | 30% | 20% |
| Switzerland | 1.00 | 0.50 | 184.2 | 197.8 | 2.91 | 2.92 | 34% | 17% |
| Turkey | 2.39 | 2.83 | 15,505.2 | 15,238.4 | 4.04 | 3.85 | 59% | 74% |
| UK | 0.36 | 0.31 | 4,253.0 | 4,627.5 | 2.63 | 2.62 | 13% | 12% |
| Ukraine | 0.64 | 0.40 | 23,132.0 | 29,823.0 | 2.18 | 2.06 | 29% | 20% |
| Europe | 0.98 | 0.90 | 127,361.0 | 141,512.1 | 2.76 | 2.61 | 35% | 34% |

Supply Density = available honeybee colonies per hectare of insect pollinated crop, Total Demand = total number of colonies demanded under average RSR assumptions. Density of demand = mean number of colonies required per hectare divided by two to represent colony movement within the year. Pollination service capacity = maximum capacity of honeybee stocks to supply pollination services, taken as supply density divided by density of demand under average recommended stocking rate assumptions
